# Trends in pancreatic cancer incidence, prevalence, and survival outcomes by histological subtypes: a retrospective cohort study

**DOI:** 10.1093/gastro/goaf030

**Published:** 2025-04-09

**Authors:** Sen Lei, Yize Mao, Qiuxia Yang, Honghong Yan, Jun Wang

**Affiliations:** Department of Pancreatobiliary Surgery, State Key Laboratory of Oncology in South China, Guangdong Provincial Clinical Research Center for Cancer, Sun Yat-sen University Cancer Center, Guangzhou, Guangdong, P. R. China; Department of Pancreatobiliary Surgery, State Key Laboratory of Oncology in South China, Guangdong Provincial Clinical Research Center for Cancer, Sun Yat-sen University Cancer Center, Guangzhou, Guangdong, P. R. China; Department of Radiology, State Key Laboratory of Oncology in South China, Guangdong Provincial Clinical Research Center for Cancer, Sun Yat-sen University Cancer Center, Guangzhou, Guangdong, P. R. China; Department of ICU, State Key Laboratory of Oncology in South China, Guangdong Provincial Clinical Research Center for Cancer, Sun Yat-sen University Cancer Center, Guangzhou, Guangdong, P. R. China; Department of Pancreatobiliary Surgery, State Key Laboratory of Oncology in South China, Guangdong Provincial Clinical Research Center for Cancer, Sun Yat-sen University Cancer Center, Guangzhou, Guangdong, P. R. China

**Keywords:** pancreatic cancer, incidence, prevalence, survival, Joinpoint

## Abstract

**Background:**

Pancreatic cancer (PC) is a heterogeneous disease with various histological and molecular subtypes. This study aimed to provide updated epidemiological estimates, survival outcomes, and treatment information for PC based on histological subtypes in the USA.

**Methods:**

Data from the US Cancer Statistics and Surveillance, Epidemiology, and End Results (SEER)-17 databases (2000–2020) were used, including adults aged ≥20 years who were diagnosed with PC. The trends of incidence and prevalence by histological types were calculated by using the Joinpoint Regression model. Survival by histological type was analysed by using Kaplan–Meier curves and log-rank tests for group comparisons.

**Results:**

Overall, the age-adjusted PC incidence per 100,000 increased from 9.54 to 12.05 in SEER-17 and from 9.75 to 12.19 in the US Cancer Statistics between 2001 and 2019. A further SEER-17 study comprised 113,681 PC cases that were sorted by histologic type between 2000 and 2020. The incidence per 100,000 of invasive intraductal papillary mucinous neoplasm (IPMN) and invasive mucinous cystic neoplasm (MCN) decreased (IPMN from 0.67 to 0.20 and MCN from 0.05 to 0.01) whereas that of other histological subtypes increased. Survival analysis indicated the best outcomes for solid pseudopapillary tumors and the poorest for squamous cell carcinoma. At the localized stage, the proportion of surgery in the treatment modalities varied depending on the biological behavior; the proportion of surgery for pancreatic neuroendocrine tumor was the highest and that for pancreatic ductal adenocarcinoma (PDAC) was the lowest. At the distant metastasis stage, a chemotherapy-based regimen remained the primary treatment of PDAC, pancreatic neuroendocrine tumor, and IPMN.

**Conclusions:**

PC incidence and prevalence have been increasing. The incidence of IPMN and MCN decreased whereas that of other subtypes increased. Treatment distribution varies among subtypes and stages.

## Introduction

Pancreatic cancer (PC) is one of the deadliest cancers worldwide, with a 5-year overall survival of ∼10% [1, 2]. According to the GLOBOCAN 2020 report, ∼495,773 new PC cases were diagnosed worldwide, accounting for 2.6% of all cancer diagnoses [3]. In the World Health Organization classification, PC comprises several histological subtypes, such as pancreatic ductal adenocarcinoma (PDAC), invasive intraductal papillary mucinous neoplasm (IPMN), pancreatic neuroendocrine tumor (pNET), adenosquamous carcinoma (ASC), invasive mucinous cystic neoplasm (MCN), acinar cell carcinoma (ACC), squamous cell carcinoma (SCC), and solid pseudopapillary tumor (SPT). PDAC constitutes ∼90% of all primary PC cases, with other histological subtypes being less prevalent [4]. Each histological subtype possesses distinct biological behavior. For example, pNET, IPMN, MCN, SPT, and ACC display indolent characteristics whereas PDAC, ASC, and SCC exhibit aggressive tendencies [4]. The elucidation of contemporary epidemiological trends in PC according to specific histological subtypes is important to provide insights for preventive interventions and clinical management.

Much of the earlier research has shown an overall rise in PC incidence. However, these studies lack specific information on each histological subtype [3, 5]. Additionally, several studies have independently reported the incidence trends for individual PC subtypes, with the majority focusing on PDAC [5–9]. Consequently, data regarding the incidence patterns of other subtypes, namely MCN, SPT, and SCC, within the USA have remained conspicuously absent. Previous research reported the incidence of PC based on Surveillance, Epidemiology, and End Results (SEER)-9 and SEER-13 data [10]. However, these studies lack the details of MCN, SPT, and SCC, and the data were not the latest. Moreover, given the evolving treatment paradigms and advancements in therapeutic modalities, transformations in the therapeutic approach for PC across various histological subtypes remain unclear. Therefore, in this study, we aimed to comprehensively evaluate the demographics, treatment, and outcomes of PC by histological subtype by utilizing data that were sourced from the United States Cancer Statistics (USCS) and SEER-17 databases.

## Materials and methods

### Data source

We conducted our analysis by using the USCS and SEER-17 databases (version November 2022). The USCS database comprises population-based cancer registry data that are collected by the National Cancer Institute’s SEER program and the Center for Disease Control and Prevention National Program of Cancer Registries (NPCR). The NPCR supports central cancer registries in 46 states and several US territories, representing >95% of the US population [11]. The SEER program is a coordinated system of population-based state cancer registries that collects incidence and survival data on cases reported from target geographic areas. The SEER-17 data were sourced from 17 population-based cancer registries, representing ∼26.5% of the US population in 2020 [12].

### Study population

This study included patients who were aged ≥20 years and were newly diagnosed with PC that was identified by using the International Classification of Diseases for Oncology, Third Edition (ICD-O-3) codes C25.0 to C25.9. Cases were classified by histologic type (ICD-O histologic codes in Supplementary Table S1) as PDAC, IPMN, pNET, ASC, MCN, ACC, SCC, or SPT. Patients with other primary malignancies, no microscopic confirmation, unknown age, or a diagnosis that was made solely on an autopsy or death certificate were excluded. The stage at diagnosis was categorized based on the Summary Stage 2000 (2000–2003) and Combined Summary Stage (2004–2020) as localized (neoplasm confined to the pancreas), regional (neoplasm spread to the regional lymph nodes), distant (neoplasm metastasis), or unstaged/unknown. We evaluated common PC treatment methods: surgery, chemotherapy, and radiotherapy. Consequently, treatments were categorized as none, surgery, radiotherapy, chemotherapy, surgery+radiotherapy, surgery+chemotherapy, radiotherapy+chemotherapy, or surgery+radiotherapy+chemotherapy. The SEER and USCS data did not disclose sensitive patient information or identifying information. As a result, neither ethical approval nor informed patient consent was necessary.

### Statistical analysis

Age-adjusted incidence rates were calculated by using the direct method, utilizing the 2000 US standard population for reference. The average annual percentage change (AAPC) and annual percentage change (APC) were calculated to examine incidence trends. Limited-duration prevalence rates were calculated for 20 years. The survival period was calculated from the date of the final diagnosis to the date of the last follow-up or death. Survival analyses were performed by using Kaplan–Meier curves and different groups were compared by using log-rank tests. Univariate analyses of patients with different histological subtypes were performed by using Cox proportional hazards regression. Hazard ratios (HRs) and 95% confidence intervals (CIs) were calculated. The distribution of stages at diagnosis or lymph node status was compared among each histological subtype by using the chi-square test.

Analyses were performed from 20 June 2023 to 10 September 2023. Age-adjusted incidence rates and limited-duration prevalence rates were calculated by using SEER*Stat software (version 8.2.1). AAPC, APC, and 95% CIs were calculated by using Joinpoint software (Version 4.9.1.0). All other statistical analyses were performed by using R (version 4.2.1). Significance was attributed to comparative differences at a threshold of *P* < 0.05.

## Results

### Baseline characteristics

The characteristics of 113,681 patients with PC according to histologic subtypes in SEER-17 from 2000 to 2020 are presented in Table 1; Supplementary Figure S1 shows the flowchart of the study population. Among these patients, there were 96,807 (85.2%) PDAC, 9,368 (8.2%) pNET, 5,222 (4.6%) IPMN, 883 (0.8%) ASC, 391 (0.3%) ACC, 391 (0.3%) SPT, 306 (0.3%) SCC, and 313 (0.3%) MCN cases. The age–sex pyramid of PC by histologic subtypes is shown in Supplementary Figure S2. As indicated by the age–frequency distribution, PDAC, SCC, ASC, pNET, IPMN, ACC, and MCN demonstrated a unimodal skewness distribution with the peak incidence that occurred in males aged 60–79 years. Similar findings were observed in females. However, in females, SPT demonstrated a bimodal distribution with early-onset and late-onset peak incidences at ∼20–24 and ∼40–44 years.

**Table 1.** Baseline characteristics of PC by histological subtypes

Characteristic	PDAC	pNET	IPMN	ASC	SPT	ACC	MCN	SCC

**%Pancreatic cancer**	96,807 (85.2%)	9,368 (8.2%)	5,222 (4.6%)	883 (0.8%)	391 (0.3%)	391 (0.3%)	313 (0.3%)	306 (0.3%)
**Age (years)**								
<50	5,847 (6.0%)	2,121 (22.6%)	373 (7.1%)	44 (5.0%)	312 (79.8%)	54 (13.8%)	74 (23.6%)	28 (9.2%)
50–59	17,623 (18.2%)	2,307 (24.6%)	959 (18.4%)	140 (15.9%)	48 (12.3%)	82 (21.0%)	49 (15.7%)	62 (20.3%)
60–69	29,220 (30.2%)	2,614 (27.9%)	1,545 (29.6%)	278 (31.5%)	23 (5.9%)	108 (27.6%)	66 (21.1%)	65 (21.2%)
70–79	28,197 (29.1%)	1,721 (18.4%)	1,530 (29.3%)	296 (33.5%)	6 (1.5%)	99 (25.3%)	73 (23.3%)	90 (29.4%)
80+	15,920 (16.4%)	605 (6.5%)	815 (15.6%)	125 (14.2%)	2 (0.5%)	48 (12.3%)	51 (16.3%)	61 (19.9%)
**Sex**								
Male	50,271 (51.9%)	5,085 (54.3%)	2,514 (48.1%)	463 (52.4%)	57 (14.6%)	277 (70.8%)	78 (24.9%)	164 (53.6%)
Female	46,536 (48.1%)	4,283 (45.7%)	2,708 (51.9%)	420 (47.6%)	334 (85.4%)	114 (29.2%)	235 (75.1%)	142 (46.4%)
**Race**								
White	76,837 (79.4%)	7,356 (78.5%)	4,176 (80.0%)	720 (81.5%)	263 (67.3%)	319 (81.6%)	244 (78.0%)	220 (71.9%)
Black	11,535 (11.9%)	1,074 (11.5%)	567 (10.9%)	81 (9.2%)	69 (17.6%)	36 (9.2%)	38 (12.1%)	44 (14.4%)
Others	8,148 (8.4%)	870 (9.3%)	458 (8.8%)	78 (8.8%)	56 (14.3%)	35 (9.0%)	30 (9.6%)	39 (12.7%)
Unknown	287 (0.3%)	68 (0.7%)	21 (0.4%)	4 (0.5%)	3 (0.8%)	1 (0.3%)	1 (0.3%)	3 (1.0%)
**Location**								
Head	48,098 (49.7%)	2,771 (29.6%)	2,316 (44.4%)	385 (43.6%)	103 (26.3%)	173 (44.2%)	86 (27.5%)	126 (41.2%)
Body/Tail	25,296 (26.1%)	4,345 (46.4%)	1,406 (26.9%)	319 (36.1%)	228 (58.3%)	117 (29.9%)	157 (50.2%)	106 (34.6%)
Others	23,413 (24.2%)	2,252 (24.0%)	1,500 (28.7%)	179 (20.3%)	60 (15.3%)	101 (25.8%)	70 (22.4%)	74 (24.2%)
**Year of diagnosis**								
2000–2006	24,919 (25.7%)	1,370 (14.6%)	2,076 (39.8%)	184 (20.8%)	38 (9.7%)	90 (23.0%)	169 (54.0%)	67 (21.9%)
2007–2013	31,990 (33.0%)	2,804 (29.9%)	1,754 (33.6%)	272 (30.8%)	73 (18.7%)	119 (30.4%)	99 (31.6%)	103 (33.7%)
2014–2020	39,898 (41.2%)	5,194 (55.4%)	1,392 (26.7%)	427 (48.4%)	280 (71.6%)	182 (46.5%)	45 (14.4%)	136 (44.4%)
**Stage**								
Localized	7,141 (7.4%)	3,504 (37.4%)	548 (10.5%)	70 (7.9%)	263 (67.3%)	54 (13.8%)	124 (39.6%)	17 (5.6%)
Regional	28,933 (29.9%)	1,691 (18.1%)	1,419 (27.2%)	350 (39.6%)	86 (22.0%)	116 (29.7%)	98 (31.3%)	87 (28.4%)
Distant	57,349 (59.2%)	3,928 (41.9%)	3,127 (59.9%)	450 (51.0%)	28 (7.2%)	204 (52.2%)	78 (24.9%)	195 (63.7%)
Unknown/unstaged	3,384 (3.5%)	245 (2.6%)	128 (2.5%)	13 (1.5%)	14 (3.6%)	17 (4.3%)	13 (4.2%)	7 (2.3%)
**Grade**								
I/II	14,273 (53.6%)	4,682 (88.4%)	1,435 (71.1%)	112 (27.8%)	68 (97.1%)	45 (45.0%)	114 (74.5%)	26 (23.9%)
III/IV	12,348 (46.4%)	612 (11.6%)	584 (28.9%)	291 (72.2%)	2 (2.9%)	55 (55.0%)	39 (25.5%)	83 (76.1%)
**Surgery**								
Yes	12,749 (13.2%)	4,771 (50.9%)	1,317 (25.2%)	321 (36.4%)	347 (88.7%)	126 (32.2%)	207 (66.1%)	25 (8.2%)
No/unknown	84,058 (86.8%)	4,597 (49.1%)	3,905 (74.8%)	562 (63.6%)	44 (11.3%)	265 (67.8%)	106 (33.9%)	281 (91.8%)
**Chemotherapy**								
Yes	53,379 (55.1%)	2,169 (23.2%)	2,688 (51.5%)	526 (59.6%)	16 (4.1%)	236 (60.4%)	106 (33.9%)	150 (49.0%)
No/unknown	43,428 (44.9%)	7,199 (76.8%)	2,534 (48.5%)	357 (40.4%)	375 (95.9%)	155 (39.6%)	207 (66.1%)	156 (51.0%)
**Radiotherapy**								
Yes	14,758 (15.2%)	379 (4.0%)	747 (14.3%)	131 (14.8%)	8 (2.0%)	50 (12.8%)	51 (16.3%)	37 (12.1%)
No/unknown	82,049 (84.8%)	8,989 (96.0%)	4,475 (85.7%)	752 (85.2%)	383 (98.0%)	341 (87.2%)	262 (83.7%)	269 (87.9%)
**Lymph node metastasis**								
Yes	24,028 (36.0%)	2,199 (28.9%)	1,244 (36.1%)	313 (46.4%)	19 (5.2%)	116 (39.6%)	61 (23.8%)	83 (42.8%)
No	42,740 (64.0%)	5,400 (71.1%)	2,198 (63.9%)	362 (53.6%)	347 (94.8%)	177 (60.4%)	195 (76.2%)	111 (57.2%)

All data are presented as number of cases followed by percentage in parentheses.

### Incidence

**Figure 1.**
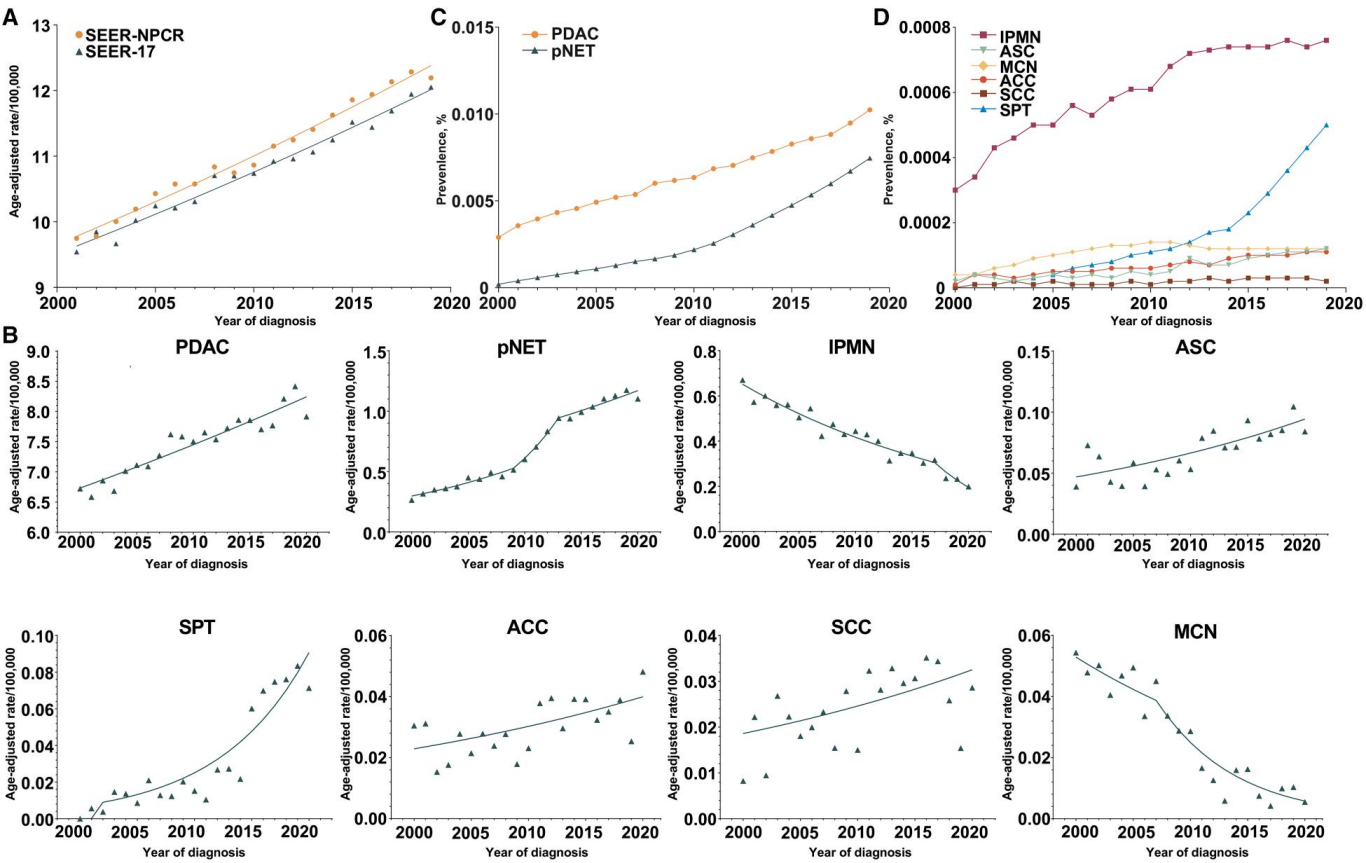
Annual incidence and overall PC prevalence by histologic subtypes. (A) Overall PC incidence (SEER-NPCR; SEER-17, 2001–2019). (B) Annual PC incidence by histologic subtypes (SEER-17, 2000–2020). (C) PDAC and pNET prevalence. (D) IPMN, ASC, MCN, ACC, SCC, and SPT prevalence.

The SEER-NPCR showed that the overall age-adjusted incidence of PC increased from 9.75 to 12.19 per 100,000 from 2010 to 2019 (AAPC = 1.3; 95% CI, 1.2–1.4; *P *<* *0.001) (Figure 1A). In accordance with the incidence rate trends, the overall age-adjusted incidence of PC in SEER-17 increased from 9.54 to 12.05 per 100,000 over the same period (AAPC = 1.2; 95% CI, 1.2–1.3; *P *<* *0.001).

Figure 1B and Table 2 illustrate the age-adjusted incidence rates according to histological subtypes from 2000 to 2020. Overall, PDAC, pNET, ASC, ACC, and SCC showed increasing trends whereas cystic tumors, such as IPMN and MCN, showed declining trends. PDAC had the highest incidence, increasing from 6.72 to 7.92 per 100,000 person-years from 2000 to 2020 (AAPC = 1.0; 95% CI, 0.8–1.2; *P *<* *0.001). pNET had the second-highest incidence, increasing from 0.26 to 1.10 per 100,000 person-years from 2000 to 2020 (AAPC = 7.1; 95% CI, 5.4–8.8; *P *<* *0.001). The age-adjusted incidence of IPMN decreased rapidly from 0.67 to 0.20 per 100,000 person-years from 2000 to 2020 (AAPC = −5.9; 95% CI, −7.6 to −4.1; *P *<* *0.001). The age-adjusted incidence of ASC increased from 0.04 to 0.08 per 100,000 person-years between 2000 and 2020 (AAPC = 3.6; 95% CI, 2.1–5.0; *P *<* *0.001). The age-adjusted incidence of SPT increased from 0.00 to 0.07 per 100,000 person-years from 2000 to 2020 (AAPC = 69.4; 95% CI, 24.2–131.1; *P *=* *0.001). The age-adjusted incidence of ACC increased from 0.03 to 0.05 per 100,000 person-years from 2000 to 2020 (AAPC = 2.8; 95% CI, 1.1–4.6; *P *=* *0.003). The age-adjusted incidence of SCC increased from 0.01 to 0.03 per 100,000 person-years between 2000 and 2020 (AAPC = 2.8; 95% CI: 0.5–5.2; *P *=* *0.017). The age-adjusted incidence of MCN decreased from 0.05 to 0.01 per 100,000 person-years from 2000 to 2020 (AAPC = −10.5; 95% CI, −13.8 to −7.1; *P *<* *0.001).

**Table 2.** Trends in PC incidence rates (2000–2020): SEER-17

Characteristic	Overall		Joinpoint 1			Joinpoint 2			Joinpoint 3		
	AAPC (95% CI)	*P*	Year	APC (95% CI)	*P*	Year	APC (95% CI)	*P*	Year	APC (95% CI)	*P*
Histologic types											
PDAC	1.0 (0.8 to 1.2)	**<0.001**	–	–	–	–	–	–	–	–	–
pNET	−7.1 (5.4 to 8.8)	**<0.001**	2000–2009	6.6 (4.6 to 8.7)	**<0.001**	2009–2013	15.7 (7.6 to 24.4)	**0.001**	2013–2020	3.1 (1.5 to 4.7)	**0.001**
MCN	−10.5 (−13.8 to −7.1)	**<0.001**	2000–2007	−4.4 (−11.2 to 3.0)	0.220	2007–2020	−13.6 (–17.7 to −9.4)	**<0.001**	–	–	–
IPMN	−5.9 (−7.6 to −4.1)	**<0.001**	2000–2017	−4.4 (−5.0 to –3.7)	**<0.001**	2017–2020	−13.9 (−24.2 to −2.2)	**0.024**	–	–	–
SCC	2.8 (0.5 to 5.2)	**0.017**	–	–	**–**	–	–	–	–	–	–
SPT	69.4 (24.2 to 131.1)	**0.001**	2000–2002	6,031.7 (116.6 to 173,508.4)	**0.019**	2002–2020	13.7 (10.1 to 17.5)	**<0.001**	–	–	–
ASC	3.6 (2.1 to 5.0)	**<0.001**	–	–	–	–	–	–	–	–	–
ACC	2.8 (1.1 to 4.6)	**0.003**	–	–	–	–	–	–	–	–	–
Stage											
PDAC											
Localized	4.4 (3.3 to 5.6)	**<0.001**	2000–2015	2.6 (1.6 to 3.6)	**<0.001**	2015–2020	10.3 (6.4 to 14.3)	**<0.001**	–	–	–
Regional	0.8 (0.2 to 1.4)	**0.011**	2000–2011	2.0 (1.1 to 2.9)	**<0.001**	2011–2020	−0.7 (−1.7 to 0.3)	0.163	–	–	–
Distant	1.1 (0.8 to 1.4)	**<0.001**	2000–2009	1.6 (1.0 to 2.3)	**<0.001**	2009–2020	0.7 (0.3 to 1.1)	**0.002**	–	–	–
pNET											
Localized	15.1 (11.8 to 18.5)	**<0.001**	2000–2009	13.7 (8.8 to 18.9)	**<0.001**	2009–2013	36.3 (20.8 to 53.8)	**<0.001**	2013–2020	6.1 (4.0 to 8.3)	**<0.001**
Regional	6.7 (5.0 to 8.4)	**<0.001**	2000–2015	10.3 (8.7 to 12.0)	**<0.001**	2015–2020	−3.5 (−8.3 to 1.5)	0.157	–	–	–
Distant	3.5 (2.2 to 4.8)	**<0.001**	2000–2011	5.3 (3.4 to 7.3)	**<0.001**	2011–2020	1.3 (−0.5 to 3.2)	0.150	–	–	–
IPMN											
Localized	0.2 (−1.1 to 1.5)	0.770	–	–	**–**	–	–	**–**	–	–	–
Regional	−4.2 (−5.2 to −3.1)	**<0.001**	–	–	–	–	–	–	–	–	–
Distant	−7.3 (−9.8 to −4.9)	**<0.001**	2000–2017	−5.2 (−6.1 to −4.3)	**<0.001**	2017–2020	−18.7 (−32.3 to −2.2)	**0.030**	–	–	–
ASC											
Localized	8.9 (−1.0 to 19.8)	0.076	–	–	–	–	–	–	–	–	–
Regional	3.6 (1.3 to 5.9)	**0.004**	–	–	–	–	–	–	–	–	–
Distant	2.8 (1.1 to 4.6)	**0.003**	–	–	–	–	–	–	–	–	–

Statistically significant *P*-values with a level of significance set at <0.05 are highlighted in bold.

The stage-specific age-adjusted incidences of the four common PCs are shown in Supplementary Figure S3 and Table 2. For PDAC, the distant stage had the highest age-adjusted incidence, followed by regional and localized stages. The age-adjusted incidence of the localized stage had the largest growth trend (AAPC = 4.4; 95% CI, 3.3–5.6; *P *<* *0.001) followed by the distant (1.1%/year) and regional (0.8%/year) stages. For pNET, the localized stage had the largest growth trend and became the highest age-adjusted incidence (AAPC = 15.1; 95% CI, 11.8–18.5; *P *<* *0.001). Annually, distant and regional pNET cases increased by 3.5% and 6.7%, respectively. Distant and regional IPMN cases also decreased rapidly by 7.3% and 4.2% per year, respectively, whereas localized cases remained stable (AAPC = 0.2; 95% CI, −1.1 to 1.5; *P *=* *0.770). ASC cases showed an increase at all stages: localized (8.9%/year), regional (3.6%/year), and distant (2.8%/year).

### Prevalence

Reflecting the incidence and survival outcome of PC, the 20-year duration prevalence from 2000 to 2019 was calculated. The prevalence of PDAC increased slightly from 2.9 per 100,000 in 2000 to 10.24 per 100,000 in 2019 (Figure 1C). The prevalence of pNET was 0.2 per 100,000 in 2000 and increased to 7.46 per 100,000 in 2019, as estimated by averaging the 2019 and 2020 populations. The prevalence of IPMN increased from 0.3 per 100,000 in 2000 to 0.76 per 100,000 in 2019 (Figure 1D). The prevalence of SPT increased from 0.0 per 100,000 in 2000 to 0.5 per 100,000 in 2019. However, the prevalence of the other four PC subtypes did not change remarkably.

### Survival

**Figure 2.**
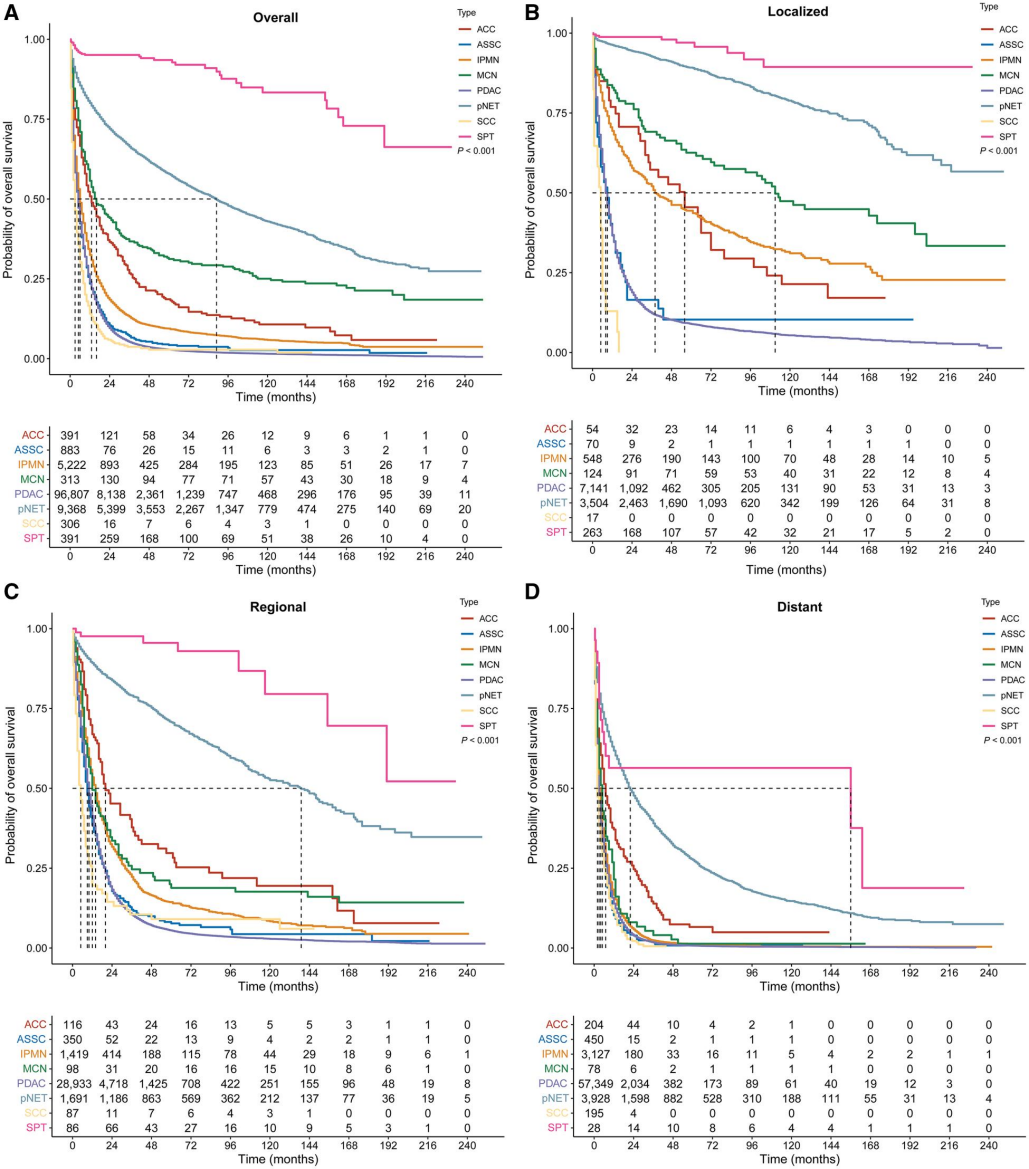
Kaplan–Meier survival curves for PC by histological subtypes in overall study population. (A) Overall survival for all stages. (B) Overall survival for localized stage. (C) Overall survival for regional stage. (D) Overall survival for distant stage.

The median overall survival (mOS) and the survival rates of PC according to histologic subtype at different stages are presented in Supplementary Table S2. Overall, patients with SCC had worse outcomes than those with PDAC (mOS, 3.0 vs 5.0 months; HR, 1.27; *P *<* *0.001; Supplementary Table S3 and Figure 2A). Patients with ASC (mOS, 5.0 months; HR, 0.95; *P *=* *0.117) had prognoses that were similar to those with PDAC. Patients with SPT (mOS, unreached; HR, 0.03; *P *<* *0.001), pNET (mOS, 89.0 months; HR, 0.17; *P *<* *0.001), MCN (mOS, 16.0 months; HR, 0.34; *P *<* *0.001), ACC (mOS, 13.0 months; HR, 0.48; *P *<* *0.001), or IPMN (mOS, 6.0 months; HR, 0.74; *P *<* *0.001) had a survival advantage over patients with PDAC. Similar findings were observed for the patients with localized, regional, and distant stages of PC (Figure 2B–D).

Based on the outcome of each PC subtype, we classified them into three biological subtypes. Specifically, pNET, MCN, and SPT were indolent, IPMN and ACC were moderate, and PDAC, ASC, and SCC were aggressive subtypes. In addition, patients with moderate or indolent subtypes had a survival advantage over those with the aggressive subtypes in the overall, localized, regional, and distant stages (*P *<* *0.05, Supplementary Figure S4).

Further survival analysis of the aggressive subtypes showed that ASC and PDAC share similar outcomes at all stages (*P *>* *0.05). ASC and PDAC had better outcomes than SCC, separately in the overall, localized, and regional stages (*P *<* *0.05). However, at the distant stage, there were no significant differences in OS between ASC, PDAC, and SCC (Supplementary Figure S5).

### Distribution of stages and lymph node metastases

More than 50% of patients with the aggressive subtypes, including PDAC, ASC, and SCC, were diagnosed at the distant stage whereas <50% of patients with the indolent subtypes, including pNET, SPT, and MCN, were diagnosed at the distant stage. Moreover, <10% of patients with the aggressive subtypes were diagnosed at the localized stage whereas >30% of patients with the indolent subtype were diagnosed at this stage (Supplementary Figure S6A and Table 1). In addition, >40% of patients with the aggressive subtype had lymph node metastasis whereas <30% of patients with the indolent subtype had lymph node metastasis (Supplementary Figure S6B and Table 1). The distribution of stages at diagnosis and lymph node status among each histological subtype was significantly different (*P *<* *0.001).

### Treatment distribution

**Figure 3.**
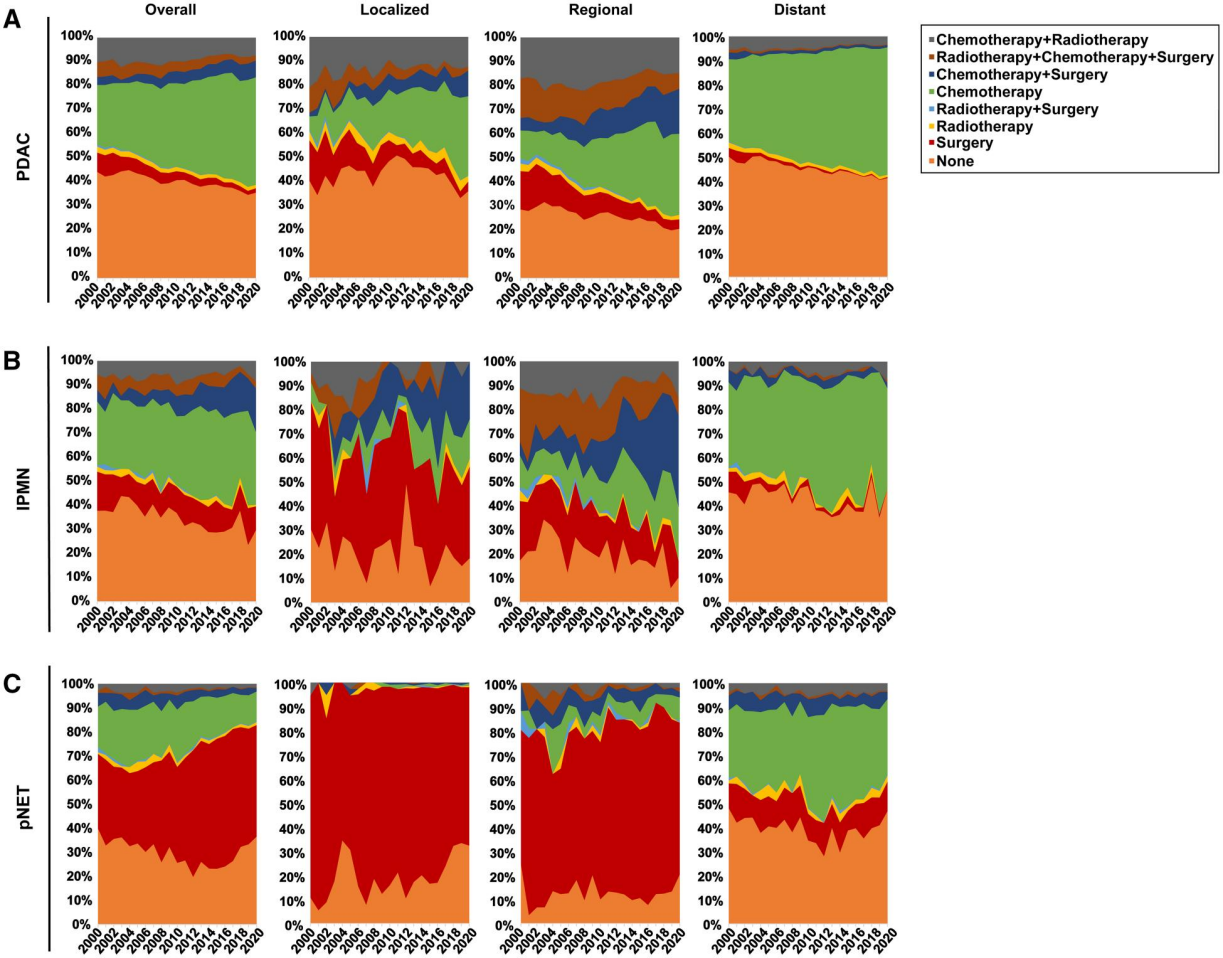
Distribution of treatment modalities by histologic subtype at different stages during 2000–2020. (A) The distribution of treatment modalities for PDAC. (B) Distribution of treatment modalities for IPMN. (C) The distribution of treatment modalities for pNET.

The distribution of treatment modalities differed according to histological subtypes (Figure 3). From 2000 to 2020, for PDAC, overall, a decrease was observed in patients who were not receiving any treatment (44.2% to 35.6%), an increase in patients who were receiving chemotherapy (24.8% to 44.7%), a decrease in patients who were receiving surgery alone (8.0% to 1.8%), and an increase in patients who were receiving surgery+chemotherapy (3.3% to 6.9%), and this distribution was similar at all stages assessed (Figure 3A and Supplementary Table 4). For IPMN, the choice of treatment modalities showed significant variation over the years, particularly in localized and regional cases. Notably, chemotherapy emerged as the predominant treatment approach for distant-stage cases (41.8% in 2020, Figure 3B and Supplementary Table 5). In contrast, for pNET, surgery remained the main treatment modality across all stages, except for distant-stage cases (Figure 3C and Supplementary Table 6). Chemotherapy is rarely used in localized-stage cases (<2%) but is widely used in distant-stage cases (>25%).

### Survival outcomes for surgery in indolent subtypes of PC at distant stages

Patients with the pNET at distant stages who underwent surgery had significantly better OS than those who did not undergo surgery (Supplementary Figure S7A) (*P *<* *0.001). A similar pattern was observed in patients with SPT (*P *=* *0.001) (Supplementary Figure S7B) and MCN (*P *=* *0.018) (Supplementary Figure S7C) at distant stages.

## Discussion

PC is a complex tumor with several histological subtypes. In this population-based study, PDAC (85.2% in SEER-17) represented the majority of primary PCs. The second-most common subtype was pNET, followed by IPMN, whereas other subtypes were uncommon. The overall trend in PC incidence demonstrated an increase from 2001 to 2019.

Based on the outcomes and biological behavior, we classified the histological subtypes of PC into three groups: indolent, moderate, and aggressive. The aggressive subtype encompassed PDAC, SCC, and ASC, with mOS ranging from 3.0 to 5.0 months. The moderate subtype comprised IPMN and ACC, with mOS ranging from 6.0 to 13.0 months. Furthermore, the indolent subtype encompassed SPT, pNET, and MCN, with mOS of >16.0 months. More patients with the aggressive rather than indolent subtype were diagnosed at the distant stage. Moreover, lymph node metastasis occurred in 36.0%–46.4% of the aggressive subtype compared with 5.2%–28.9% in the indolent subtype. These findings underscore the heterogeneity of PC and suggest individualized treatment approaches that are tailored to distinct biological and behavioral categories.

Among the aggressive subtypes, ASC has previously been identified as more aggressive than PDAC [13]. However, our study found that ASC and PDAC shared similar outcomes at all stages and similar stage distribution. Moreover, our data indicate that SCC presented a worse prognosis than ASC and PDAC at the localized stage, showing that SCC may be more aggressive than ASC and PDAC. Consistently with our findings, Makarova-Rusher *et al.* showed that patients with SCC exhibited a higher proportion of poorly differentiated histology than those with PDAC [14]. Given that the median OS of SCC is 5.0 months at the localized and regional stages and 2.0 months at the distant stage, more attempts should be made to improve the outcomes of these patients. Notably, the trends in incidence analysis showed that the age-adjusted incidence of each aggressive subtype increased. When examined according to stage, we observed an increased incidence of ASC and PDAC at all stages. Considering that the localized stage had better outcomes than the regional and distant stages of the aggressive subtype, early detection of these aggressive subtypes may be beneficial to enhance treatment and improve the quality of life of the patient.

Timely and effective detection and monitoring of precancerous conditions play a pivotal role in early cancer detection, potentially preventing cancer development [15]. The international consensus guidelines for IPMN and MCN [16] recommend the resection of all MCNs unless contraindications for surgery exist due to the tendency of MCNs to progress to malignancy, often with low resectability and poor prognoses. Similarly, for IPMN, the guidelines advocate resection of the main duct and mixed-variant IPMNs in cases in which patients are viable surgical candidates with reasonable life expectancies. Thus, timely resection of MCN and IPMN may contribute to a decrease in their incidence. Consequently, we observed a reduction in the incidence of MCN and IPMN. When analysed by stage, a decline in the incidence of IPMN was observed at the regional and distant stages. Thus, the recommendation for the resection of high-risk IPMN and MCN may cause a significant decrease in the incidence of IPMN and MCN, highlighting the importance of early interventions for precancerous lesions in preventing cancer occurrence.

Despite the favorable outcomes of indolent subtypes compared with aggressive or moderate subtypes, the age-adjusted incidence of SPT and pNET continued to rise. The increase in the age-adjusted incidence of pNET was consistent with previous studies [9, 17]. Additionally, we noted an increase in the incidence of SPT from 2000 to 2020, which few studies have reported. Moreover, previous studies showed that, even in locally advanced or metastatic disease or after re-resection of recurrent disease, a yearly lifelong follow-up is mandatory if the patient (patients with SPT or pNET) undergoes surgery [18]. Additionally, various studies have demonstrated that excisional therapy for primary and distant lesions can enhance outcomes [19]. Interestingly, our study also identified surgery as a potential means of extending the survival of patients with SPT, pNET, and MCN at the distant stage. This finding underscores the potential benefit of adopting an aggressive surgical approach to improve outcomes in advanced stages of SPT, pNET, and MCN.

In addition, given the distinct biological behaviors that were observed across histological subtypes, treatment approaches vary. Overall, surgery was the most prominent in pNET of indolent subtype, followed by IPMN in moderate subtype and PDAC in aggressive subtype. Moreover, surgery remains the main treatment for pNET, even in cases at the regional stages. In addition, chemotherapy-based treatment remains the primary treatment for pNET, and IPMN at distant stages and all stages of PDAC. Due to advancements in drug discovery for PDAC [20], we also observed an increasing use of chemotherapy at the distant stage. These findings underscore the need to diversify treatment strategies that are tailored to distinctive biological behavior types, particularly in cases that present at the localized stage.

Recently, several potential confounding factors have likely contributed to the observed changes in the incidence rates, survival outcomes, and treatment patterns of PC. First, modifications in screening practices have played a pivotal role. Enhanced screening protocols have led to earlier detection, which can improve survival rates and alter treatment approaches. Additionally, advancements in imaging technologies, such as transabdominal ultrasound, computed tomography, magnetic resonance imaging, and positron emission tomography, have significantly improved the accuracy of PC diagnosis. These technologies enable the identification of tumors at earlier stages and facilitate more precise staging, which is crucial for treatment planning and prognosis [21]. Moreover, evolving diagnostic criteria have influenced the reported incidence rates. The histologic classification of PC has also improved since the 1990s. The refinement of these criteria has enabled more consistent and accurate diagnoses, reducing the likelihood of misclassification and underreporting [22]. This standardization is essential for reliable epidemiological assessments and comparisons over time. Improvements in data collection methods, including the integration of electronic health records and the establishment of comprehensive cancer registries, have further enhanced the quality and completeness of PC data. These advancements ensure more robust and representative data, enabling better-informed public health decisions and research conclusions.

The strengths of this study lie in the utilization of a large population-based dataset (SEER-17), which enabled the analysis of recent PC incidence rates based on histological subtypes using clinicopathological parameters. To the best of our knowledge, this is the first study to comprehensively present the trends in the incidence, prevalence, survival, and therapy of PC according to histological subtypes. However, this study had some limitations. First, the database lacked data regarding the etiology of PC, which could explain the variations in epidemiological data. Second, some tumor parameters were unavailable from SEER, potentially influencing the accuracy of the trends that were stratified by tumor characteristics. Third, the scope of this study was limited to the USA and its findings may not be applicable or generalizable to other countries or people. Therefore, further studies should be conducted in different countries and healthcare systems to validate our findings. Fourth, this study does not provide a detailed analysis of the treatment outcomes associated with specific therapeutic approaches. This omission restricts our ability to draw definitive conclusions regarding the optimal treatment strategies for different subtypes and stages of PC.

## Conclusions

The study recognizes the complexity of PC, comprising several histological subtypes. This insight emphasizes the need for personalized approaches in both research and clinical management. PDAC overwhelmingly represents the majority of primary PCs. Despite better outcomes, the increasing incidence of indolent subtypes, particularly pNET and SPT, raises questions about the factors that are contributing to this trend. Treatment approaches vary across histological subtypes, emphasizing the importance of tailored strategies. Surgery remains prominent for pNET whereas chemotherapy dominates for PDAC, pNET, and IPMN at distant stages.

## Ethical approval

Not required. The SEER and USCS data did not disclose sensitive patient information or identifying information. As a result, neither ethical approval nor informed patient consent was necessary.

## Supplementary Material

goaf030_Supplementary_Data

## Data Availability

The data sets that were generated and analyzed during this study are available in the US Cancer Statistics (https://www.cdc.gov/cancer/uscs/) and Surveillance, Epidemiology, and End Results database (https://seer.cancer.gov/).
