# Supplementary material for: Trends in pancreatic cancer incidence, prevalence, and survival outcomes by histological subtypes: a retrospective cohort study
Source: Gastroenterol Rep (Oxf). 2025 Apr 9;13:goaf030. doi: 10.1093/gastro/goaf030 (PMC11981714; doi:10.1093/gastro/goaf030)
Supplement: goaf030_Supplementary_Data [file goaf030_supplementary_data.zip › supplementary figures.docx]

**Supplementary Figure S1.** The flowchart of the study population.


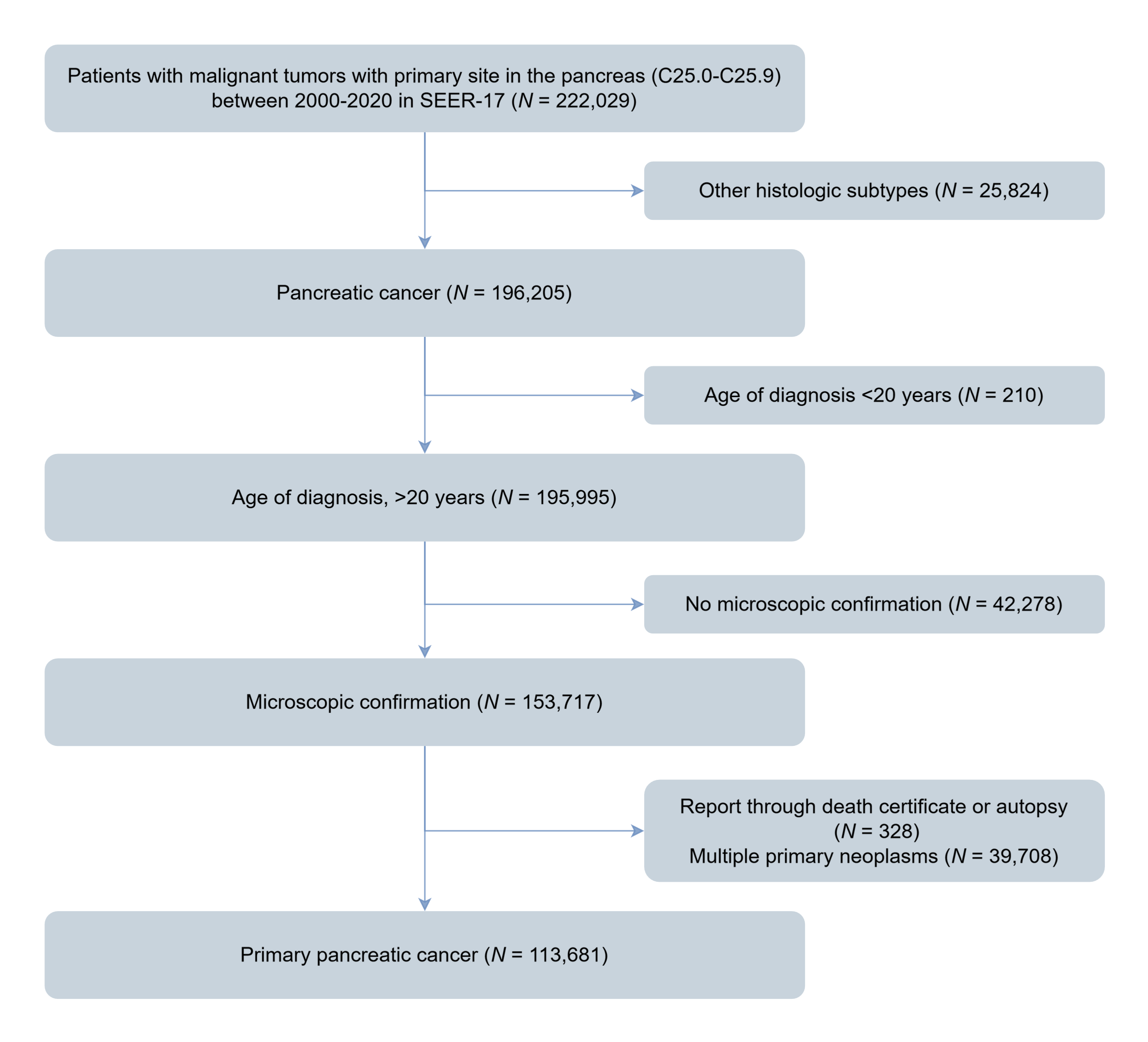


**Supplementary Figure S2.** Age-sex pyramid of pancreatic cancer by histologic subtypes.


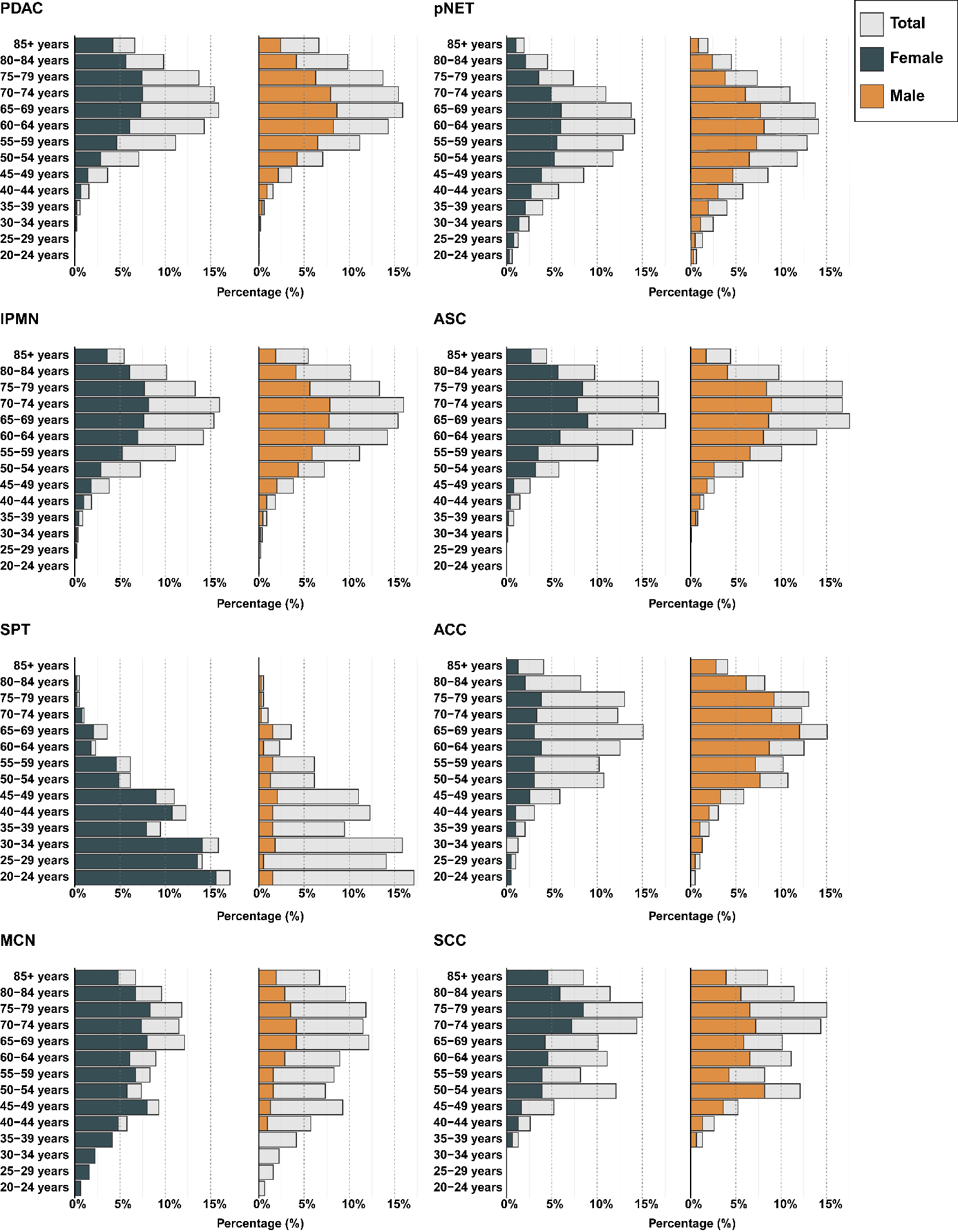


**Supplementary Figure S3.** PC incidence according to histological subtype at different stages. A, Incidence of PDAC according to stage. B, Incidence of pNET according to stage. C, Incidence of IPMN according to stage. D, Incidence of ASC according to stage.


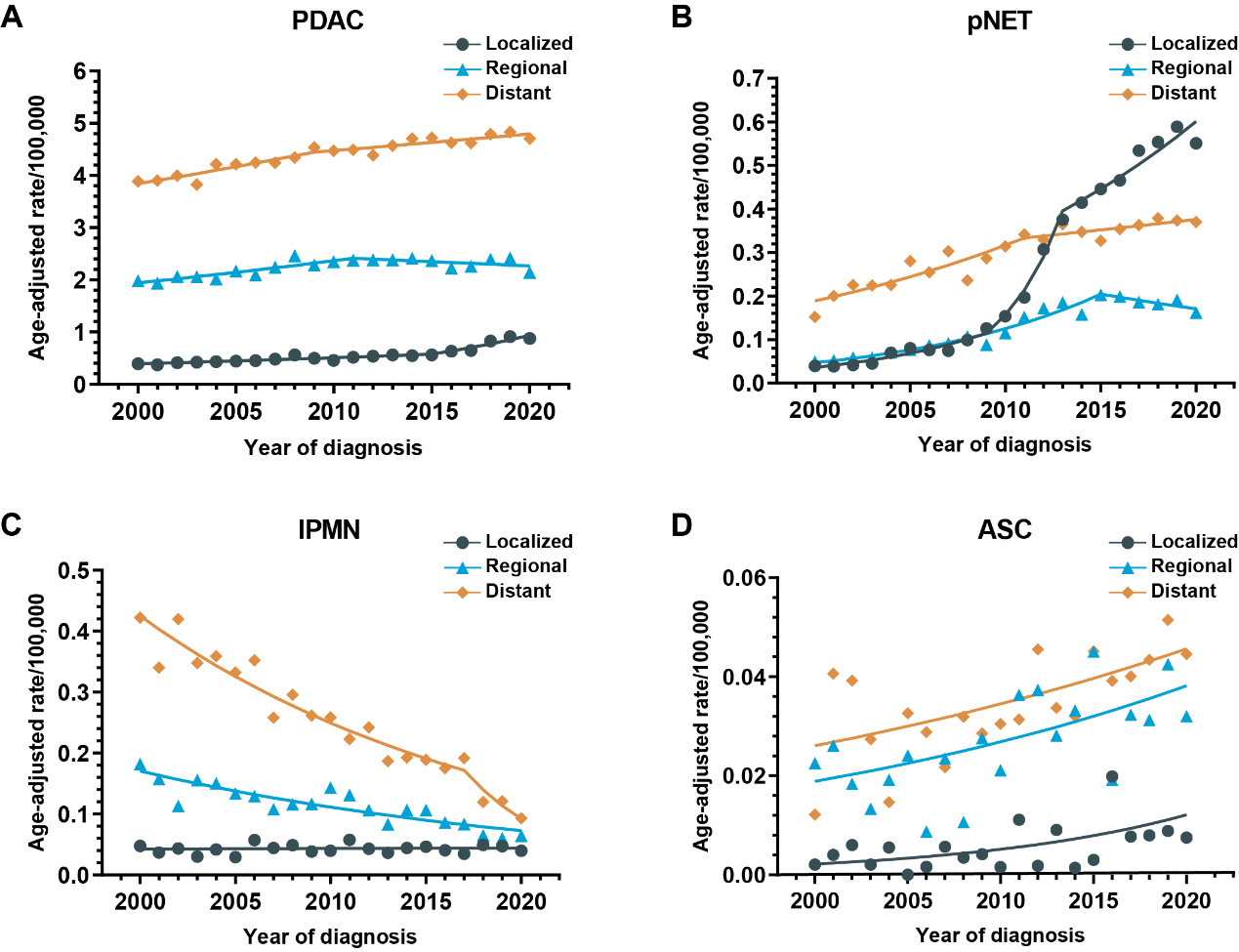


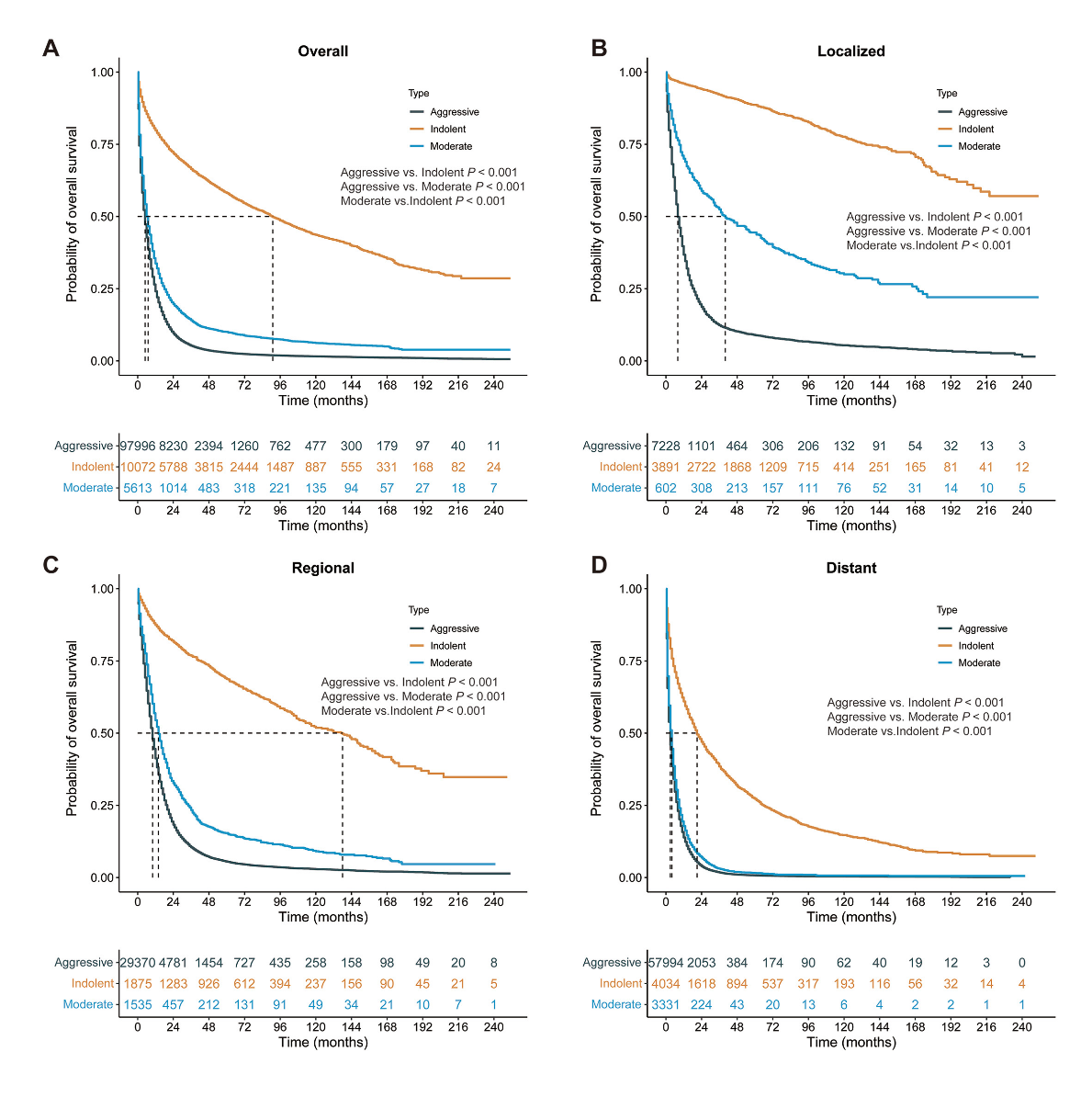
**Supplementary Figure S4.** Kaplan–Meier survival curves for PC according to biological behavior subtypes in the overall study population. A, Overall survival at all stages. B, Overall survival at localized stage. C, Overall survival at regional stage. D, Overall survival at distant stage.

**Supplementary Figure S5.** Kaplan–Meier survival curves for aggressive PC subtypes according to histological subtype in the overall study population. A, Overall survival at all stages. B, Overall survival at localized stage. C, Overall survival at regional stage. D, Overall survival at distant stage.


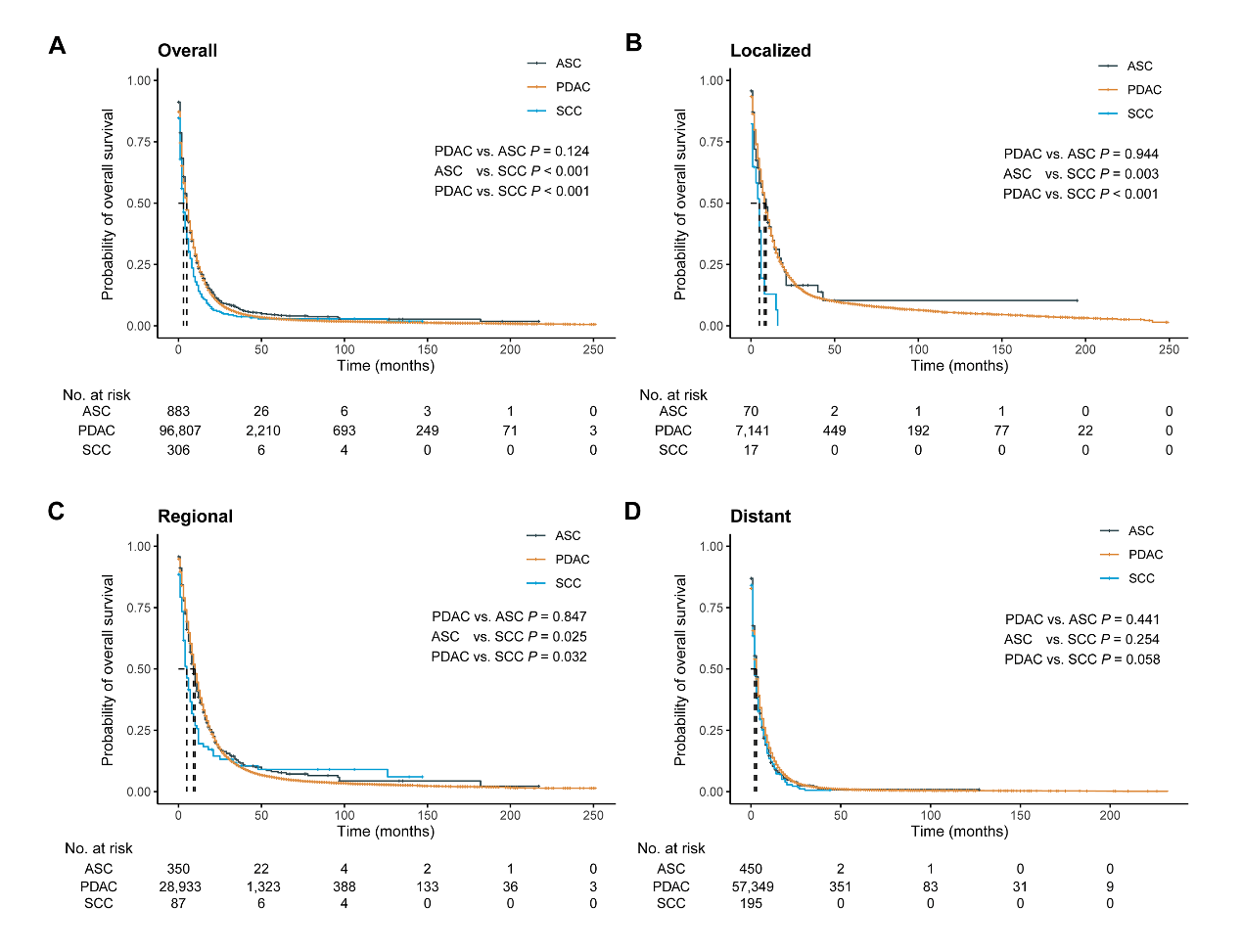


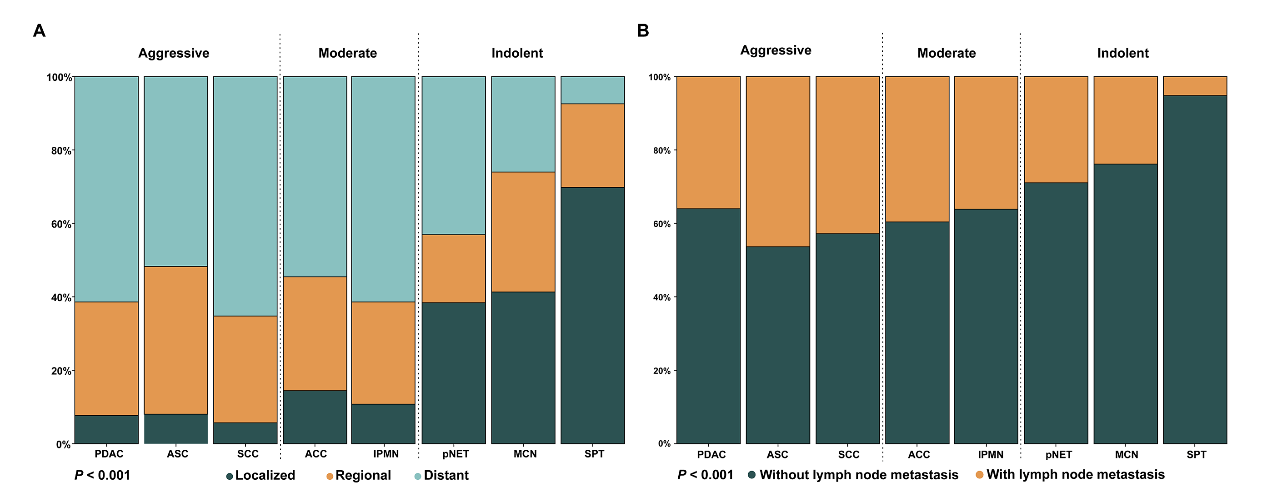
**Supplementary Figure S6.** Distribution of stage and status of lymph node metastasis according to histological subtype at diagnosis. A, Distribution of stages according to the histological subtype at diagnosis. B, Distribution of lymph node metastasis status according to histological subtype at diagnosis.


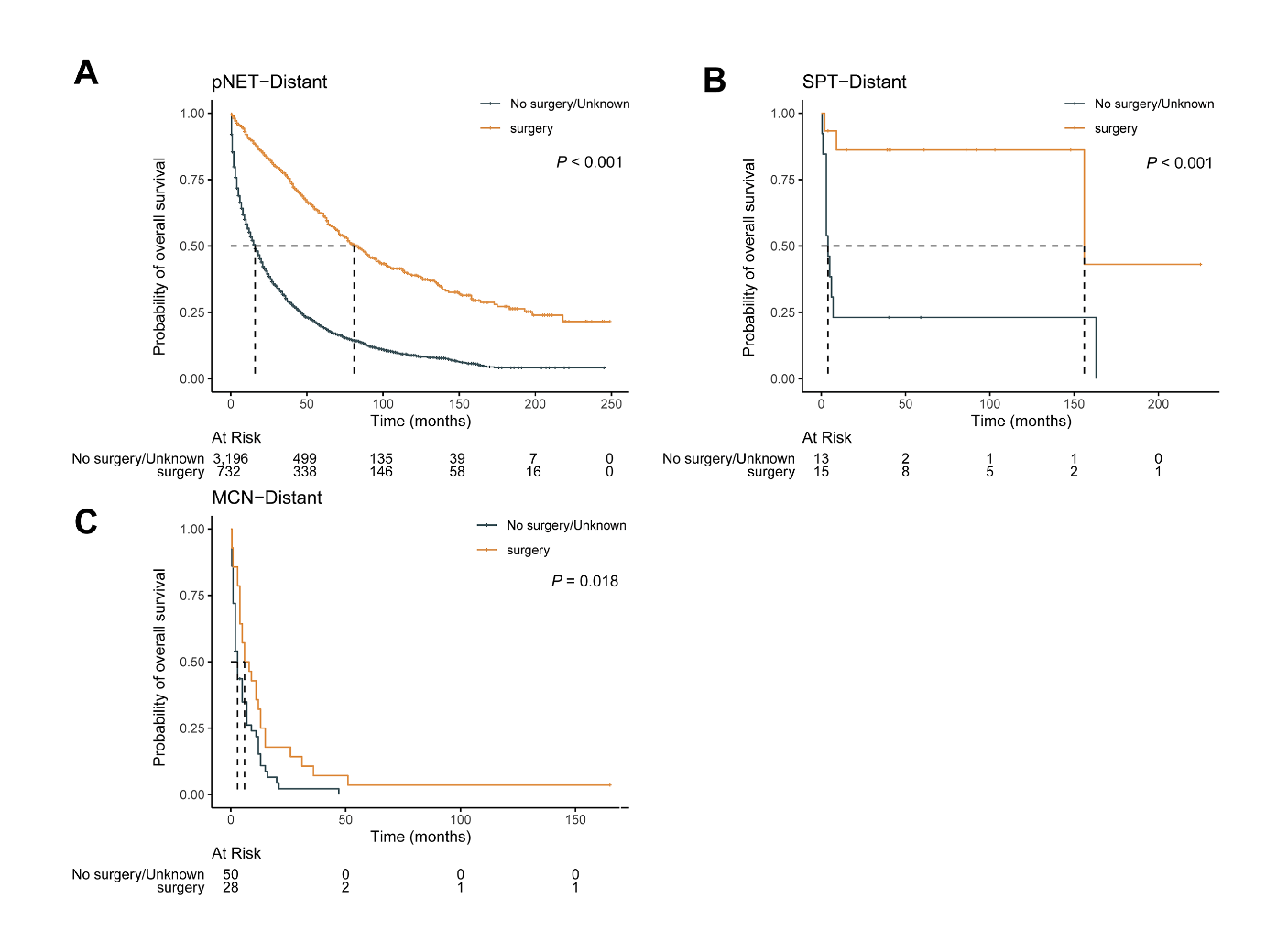
**Supplementary Figure S7.** Kaplan–Meier survival curves for indolent subtypes of PC by surgery in distant stage. A, Overall survival for pNET. B, Overall survival for SPT. C, Overall survival for MCN.
