# Supplementary material for: Trends in pancreatic cancer incidence, prevalence, and survival outcomes by histological subtypes: a retrospective cohort study
Source: Gastroenterol Rep (Oxf). 2025 Apr 9;13:goaf030. doi: 10.1093/gastro/goaf030 (PMC11981714; doi:10.1093/gastro/goaf030)
Supplement: goaf030_Supplementary_Data [file goaf030_supplementary_data.zip › 2024-213_Supplementary_tables.docx]

**Supplementary Table S1.** The ICD-O-3 codes of pancreatic cancer by histologic subtypes.

| Histologic type | ICD-O-3 Codes ^[1]^ |
| --- | --- |
| PDAC | 8140, 8141, 8142, 8143, 8144, 8145, 8146, 8147 |
| IPMN | 8050, 8260, 8450, 8453, 8471, 8480, 8481, 8503 |
| pNET | 8150, 8151, 8152, 8153, 8155, 8156, 8157, 8240, 8241, 8242, 8243, 8246, 8249 |
| ASC | 8560, 8570 |
| MCN | 8440, 8470 |
| ACC | 8550 |
| SCC | 8052, 8053, 8070, 8071, 8072, 8073, 8074, 8075, 8076, 8077, 8078, 8083, 8084 |
| SPT | 8452 |

*Abbreviations:* PDAC, pancreatic ductal adenocarcinoma; IPMN, invasive intraductal papillary mucinous neoplasm; pNET, pancreatic neuroendocrine tumor; ASC, adenosquamous carcinoma; MCN, invasive mucinous cystic neoplasm; ACC, acinar cell carcinoma; SCC, squamous cell carcinoma; SPT, solid pseudopapillary tumor

**Reference**

1.Luo G, Fan Z, Gong Y, Jin K, Yang C, Cheng H, Huang D, Ni Q, Liu C, Yu X. Characteristics and Outcomes of Pancreatic Cancer by Histological Subtypes. Pancreas. 2019 Jul;48(6):817-822.

**Supplementary Table S2.** Median overall survival, 1-year, 2-year, and 5-year survival rate of patients with pancreatic cancers by histological subtypes.

| Overall survival | PDAC | IPMN | pNET | ASC | MCN | ACC | SCC | SPT |
| --- | --- | --- | --- | --- | --- | --- | --- | --- |
| Median, months |  |  |  |  |  |  |  |  |
| Overall | 5.0 | 6.0 | 89.0 | 5.0 | 16.0 | 13.0 | 3.0 | NA^a^ |
| Localized | 8.0 | 38.0 | NA^a^ | 9.0 | 111.0 | 56.0 | 5.0 | NA^a^ |
| Regional | 10.0 | 14.0 | 139.0 | 9.0 | 12.0 | 20.0 | 5.0 | NA^a^ |
| Distant | 3.0 | 4.0 | 22.0 | 3.0 | 5.0 | 7.0 | 2.0 | 156.0 |
| 1-year survival, % |  |  |  |  |  |  |  |  |
| Overall | 24.1 | 32.5 | 80.0 | 23.5 | 55.7 | 50.9 | 14.1 | 95.1 |
| Localized | 36.8 | 69.4 | 96.3 | 36.7 | 82.9 | 76.9 | 12.9 | 98.8 |
| Regional | 41.4 | 53.5 | 89.2 | 38.5 | 49.5 | 66.9 | 19.6 | 97.6 |
| Distant | 13.9 | 16.6 | 61.8 | 10.3 | 21.5 | 37.1 | 11.2 | 56.4 |
| 2-year survival, % |  |  |  |  |  |  |  |  |
| Overall | 9.5 | 18.5 | 72.0 | 10.4 | 44.1 | 36.5 | 5.9 | 95.1 |
| Localized | 18.8 | 57.7 | 94.4 | 16.5 | 77.9 | 70.7 | 0.0 | 98.8 |
| Regional | 18.3 | 31.7 | 83.8 | 17.8 | 33.6 | 45.2 | 14.5 | 97.6 |
| Distant | 4.0 | 5.8 | 47.7 | 3.9 | 8.1 | 24.7 | 2.2 | 56.4 |
| 5-year survival, % |  |  |  |  |  |  |  |  |
| Overall | 2.8 | 9.4 | 57.9 | 4.3 | 31.2 | 18.6 | 2.8 | 93.5 |
| Localized | 8.9 | 44.4 | 89.2 | 10.3 | 62.6 | 45.5 | 0.0 | 97.0 |
| Regional | 5.5 | 14.4 | 70.7 | 7.7 | 18.8 | 29.7 | 9.0 | 95.5 |
| Distant | 0.8 | 1.3 | 27.6 | 0.8 | 1.3 | 6.6 | 0.0 | 56.4 |

^a^, not calculated

*Abbreviations:* PDAC, pancreatic ductal adenocarcinoma; IPMN, invasive intraductal papillary mucinous neoplasm; pNET, pancreatic neuroendocrine tumor; ASC, adenosquamous carcinoma; MCN, invasive mucinous cystic neoplasm; ACC, acinar cell carcinoma; SCC, squamous cell carcinoma; SPT, solid pseudopapillary tumor

**Supplementary Table S3.** Cox proportional hazards survival analysis for patients with pancreatic cancer according to histologic subtypes in different stages.

|  |  | Overall | | | Localized | | | Regional | | | Distant | | |
| --- | --- | --- | --- | --- | --- | --- | --- | --- | --- | --- | --- | --- | --- |
| Model |  | HR | CI | *p* | HR | CI | *p* | HR | CI | *p* | HR | CI | *p* |
| **Unadjusted** | Histologic types |  |  |  |  |  |  |  |  |  |  |  |  |
|  | PDAC | 1.00 (reference) | | | 1.00 (reference) | | | 1.00 (reference) | | | 1.00 (reference) | | |
|  | pNET | 0.17 | 0.16–0.17 | <0.001 | 0.06 | 0.05–0.06 | <0.001 | 0.15 | 0.13–0.16 | <0.001 | 0.26 | 0.25–0.27 | <0.001 |
|  | MCN | 0.34 | 0.30–0.38 | <0.001 | 0.22 | 0.17–0.28 | <0.001 | 0.56 | 0.45–0.70 | <0.001 | 0.76 | 0.60–0.95 | 0.016 |
|  | IPMN | 0.74 | 0.72–0.76 | <0.001 | 0.36 | 0.32–0.40 | <0.001 | 0.69 | 0.65–0.73 | <0.001 | 0.90 | 0.87–0.93 | <0.001 |
|  | SCC | 1.27 | 1.13–1.43 | <0.001 | 2.43 | 1.48–3.96 | <0.001 | 1.27 | 1.02–1.59 | 0.037 | 1.15 | 1.00–1.33 | 0.052 |
|  | SPT | 0.03 | 0.02–0.04 | <0.001 | 0.02 | 0.01–0.03 | <0.001 | 0.04 | 0.02–0.08 | <0.001 | 0.15 | 0.09–0.25 | <0.001 |
|  | ASC | 0.95 | 0.88–1.01 | 0.117 | 1.00 | 0.76–1.30 | 0.978 | 0.99 | 0.88–1.11 | 0.830 | 1.04 | 0.94–1.14 | 0.458 |
|  | ACC | 0.48 | 0.43–0.54 | <0.001 | 0.37 | 0.27–0.52 | <0.001 | 0.45 | 0.36–0.56 | <0.001 | 0.51 | 0.44–0.59 | <0.001 |
| **Model 1^a^** | Histologic types |  |  |  |  |  |  |  |  |  |  |  |  |
|  | PDAC | 1.00 (reference) | | | 1.00 (reference) | | | 1.00 (reference) | | | 1.00 (reference) | | |
|  | pNET | 0.18 | 0.18–0.19 | <0.001 | 0.07 | 0.07–0.08 | <0.001 | 0.16 | 0.15–0.17 | <0.001 | 0.28 | 0.27–0.29 | <0.001 |
|  | MCN | 0.36 | 0.32–0.41 | <0.001 | 0.29 | 0.23–0.38 | <0.001 | 0.55 | 0.44–0.69 | <0.001 | 0.77 | 0.61–0.96 | 0.023 |
|  | IPMN | 0.75 | 0.72–0.77 | <0.001 | 0.39 | 0.35–0.43 | <0.001 | 0.69 | 0.66–0.74 | <0.001 | 0.89 | 0.86–0.93 | <0.001 |
|  | SCC | 1.26 | 1.12–1.42 | <0.001 | 2.36 | 1.44–3.86 | 0.001 | 1.26 | 1.01–1.58 | 0.043 | 1.15 | 1.00–1.33 | 0.057 |
|  | SPT | 0.04 | 0.03–0.06 | <0.001 | 0.03 | 0.02–0.07 | <0.001 | 0.05 | 0.02–0.1 | <0.001 | 0.18 | 0.11–0.31 | <0.001 |
|  | ASC | 0.95 | 0.88–1.02 | 0.123 | 0.99 | 0.76–1.30 | 0.949 | 1.01 | 0.90–1.13 | 0.872 | 1.02 | 0.93–1.12 | 0.657 |
|  | ACC | 0.49 | 0.44–0.55 | <0.001 | 0.4 | 0.29–0.55 | <0.001 | 0.49 | 0.39–0.61 | <0.001 | 0.52 | 0.45–0.61 | <0.001 |
| **Model 2^b^** | Histologic types |  |  |  |  |  |  |  |  |  |  |  |  |
|  | PDAC | 1.00 (reference) | | | 1.00 (reference) | | | 1.00 (reference) | | | 1.00 (reference) | | |
|  | pNET | 0.18 | 0.17–0.19 | <0.001 | 0.13 | 0.11–0.15 | <0.001 | 0.18 | 0.16–0.20 | <0.001 | 0.27 | 0.25–0.29 | <0.001 |
|  | MCN | 0.37 | 0.31–0.45 | <0.001 | 0.43 | 0.3–0.62 | <0.001 | 0.65 | 0.5–0.86 | 0.002 | 0.62 | 0.41–0.92 | 0.018 |
|  | IPMN | 0.74 | 0.70–0.77 | <0.001 | 0.51 | 0.43–0.6 | <0.001 | 0.73 | 0.68–0.79 | <0.001 | 0.83 | 0.77–0.89 | <0.001 |
|  | SCC | 1.13 | 0.93–1.38 | 0.228 | 1.55 | 0.64–3.76 | 0.329 | 1.03 | 0.76–1.4 | 0.834 | 1.26 | 0.94–1.67 | 0.117 |
|  | SPT | 0.04 | 0.01–0.10 | <0.001 | 0.03 | 0–0.22 | 0.001 | 0.07 | 0.01–0.47 | 0.007 | 0.21 | 0.05–0.85 | 0.028 |
|  | ASC | 0.86 | 0.77–0.95 | 0.005 | 1.00 | 0.68–1.46 | 0.988 | 1.02 | 0.88–1.19 | 0.758 | 0.84 | 0.71 – 1.00 | 0.049 |
|  | ACC | 0.47 | 0.38–0.59 | <0.001 | 0.52 | 0.28–0.97 | 0.04 | 0.49 | 0.35–0.69 | <0.001 | 0.47 | 0.34–0.66 | <0.001 |
| **Model 3^c^** | Histologic types |  |  |  |  |  |  |  |  |  |  |  |  |
|  | PDAC | 1.00 (reference) | | | 1.00 (reference) | | | 1.00 (reference) | | | 1.00 (reference) | | |
|  | pNET | 0.15 | 0.14–0.15 | <0.001 | 0.11 | 0.10–0.13 | <0.001 | 0.13 | 0.12–0.15 | <0.001 | 0.24 | 0.22–0.26 | <0.001 |
|  | MCN | 0.47 | 0.39–0.57 | <0.001 | 0.51 | 0.35–0.74 | <0.001 | 0.7 | 0.53–0.92 | 0.01 | 0.94 | 0.63–1.40 | 0.745 |
|  | IPMN | 0.77 | 0.74–0.81 | <0.001 | 0.62 | 0.53–0.74 | <0.001 | 0.74 | 0.68–0.80 | <0.001 | 0.86 | 0.80–0.92 | <0.001 |
|  | SCC | 1.04 | 0.85–1.27 | 0.702 | 1.5 | 0.62–3.63 | 0.37 | 0.88 | 0.65–1.19 | 0.39 | 1.13 | 0.85–1.51 | 0.387 |
|  | SPT | 0.04 | 0.01–0.10 | <0.001 | 0.03 | 0–0.23 | 0.001 | 0.05 | 0.01–0.37 | 0.003 | 0.25 | 0.06–0.99 | 0.048 |
|  | ASC | 1.18 | 1.06–1.32 | 0.003 | 1.31 | 0.89–1.91 | 0.166 | 1.28 | 1.10–1.48 | 0.002 | 1.07 | 0.90–1.27 | 0.434 |
|  | ACC | 0.49 | 0.39–0.6 | <0.001 | 0.4 | 0.21–0.76 | 0.005 | 0.48 | 0.34–0.68 | <0.001 | 0.51 | 0.36–0.71 | <0.001 |

^a^ Model 1 adjusted for age and sex.

^b^ Model 2 adjusted for age, sex, year of diagnosis, race, tumor site and grade.

^c^ Model 3 adjusted for age, sex, year of diagnosis, race, tumor site, grade, surgery, chemotherapy and radiotherapy.

*Abbreviations*: HR, hazard ratio, CI, confidence interval, PDAC, pancreatic ductal adenocarcinoma; IPMN, invasive intraductal papillary mucinous neoplasm; pNET, pancreatic neuroendocrine tumor; ASC, adenosquamous carcinoma; MCN, invasive mucinous cystic neoplasm; ACC, acinar cell carcinoma; SCC, squamous cell carcinoma; SPT, solid pseudopapillary tumor

**Supplementary Table S4.** Distribution of treatment in PDAC.

| Stage | Year of diagnosis | Treatment | | | | | | | |
| --- | --- | --- | --- | --- | --- | --- | --- | --- | --- |
|  |  | None | Surgery | Radiotherapy | Radiotherapy+Surgery | Chemotherapy | Chemotherapy+Surgery | Radiotherapy+Chemotherapy+Surgery | Chemotherapy+Radiotherapy |
| Overall | 2000 | 44.2% | 8.0% | 2.5% | 0.8% | 24.8% | 3.3% | 6.2% | 10.1% |
|  | 2001 | 42.3% | 8.8% | 2.3% | 0.8% | 26.1% | 3.5% | 6.4% | 9.7% |
|  | 2002 | 42.9% | 9.4% | 2.1% | 0.6% | 26.2% | 3.3% | 6.6% | 8.9% |
|  | 2003 | 44.5% | 6.2% | 2.1% | 0.6% | 27.8% | 1.4% | 5.2% | 12.3% |
|  | 2004 | 45.0% | 5.4% | 2.4% | 0.3% | 28.0% | 2.4% | 5.7% | 10.7% |
|  | 2005 | 43.6% | 6.0% | 1.7% | 0.4% | 30.2% | 3.0% | 5.1% | 9.9% |
|  | 2006 | 42.7% | 4.8% | 2.1% | 0.4% | 30.9% | 3.9% | 4.7% | 10.3% |
|  | 2007 | 41.4% | 4.7% | 2.0% | 0.4% | 32.1% | 3.7% | 4.6% | 10.9% |
|  | 2008 | 39.2% | 4.8% | 1.8% | 0.3% | 32.5% | 4.2% | 5.4% | 11.7% |
|  | 2009 | 39.5% | 4.3% | 1.7% | 0.4% | 35.2% | 4.7% | 4.3% | 10.0% |
|  | 2010 | 40.8% | 3.8% | 1.8% | 0.1% | 34.7% | 5.0% | 4.1% | 9.7% |
|  | 2011 | 40.9% | 3.1% | 1.4% | 0.2% | 35.1% | 4.9% | 4.4% | 9.9% |
|  | 2012 | 39.3% | 3.5% | 1.6% | 0.2% | 37.6% | 4.5% | 4.2% | 9.1% |
|  | 2013 | 38.1% | 3.1% | 1.7% | 0.1% | 39.5% | 4.6% | 4.1% | 8.8% |
|  | 2014 | 38.7% | 3.1% | 1.5% | 0.1% | 40.3% | 5.3% | 3.4% | 7.6% |
|  | 2015 | 39.0% | 2.8% | 1.4% | 0.0% | 40.9% | 4.9% | 3.6% | 7.4% |
|  | 2016 | 37.9% | 2.0% | 1.7% | 0.1% | 43.6% | 5.5% | 2.6% | 6.7% |
|  | 2017 | 37.6% | 2.2% | 1.4% | 0.1% | 44.1% | 5.6% | 2.2% | 6.7% |
|  | 2018 | 36.4% | 2.0% | 1.7% | 0.0% | 42.0% | 6.8% | 3.1% | 8.0% |
|  | 2019 | 34.7% | 1.7% | 1.6% | 0.0% | 44.5% | 6.7% | 2.8% | 8.0% |
|  | 2020 | 35.6% | 1.8% | 1.4% | 0.0% | 44.7% | 6.9% | 2.1% | 7.4% |
| Localized | 2000 | 40.3% | 16.8% | 3.7% | 0.5% | 5.7% | 1.6% | 10.4% | 21.0% |
|  | 2001 | 34.3% | 17.8% | 1.7% | 1.5% | 11.9% | 3.2% | 11.5% | 18.2% |
|  | 2002 | 42.4% | 18.8% | 3.8% | 1.9% | 10.4% | 1.4% | 9.8% | 11.6% |
|  | 2003 | 37.6% | 13.4% | 3.2% | 1.3% | 13.1% | 1.5% | 11.5% | 18.4% |
|  | 2004 | 45.3% | 12.0% | 2.1% | 0.5% | 12.1% | 2.2% | 8.2% | 17.5% |
|  | 2005 | 46.5% | 15.1% | 3.5% | 0.8% | 13.3% | 2.7% | 7.5% | 10.7% |
|  | 2006 | 44.3% | 11.7% | 5.2% | 0.4% | 12.3% | 5.5% | 6.3% | 14.5% |
|  | 2007 | 44.5% | 9.5% | 2.9% | 0.4% | 17.8% | 5.8% | 7.2% | 11.8% |
|  | 2008 | 37.8% | 9.5% | 5.2% | 0.0% | 18.7% | 6.4% | 4.8% | 17.5% |
|  | 2009 | 44.3% | 10.7% | 2.3% | 0.4% | 16.1% | 6.9% | 5.2% | 14.0% |
|  | 2010 | 48.3% | 8.8% | 3.4% | 0.3% | 17.3% | 6.7% | 5.7% | 9.4% |
|  | 2011 | 50.8% | 4.2% | 3.7% | 0.0% | 17.2% | 6.4% | 5.1% | 12.6% |
|  | 2012 | 49.3% | 6.3% | 2.1% | 0.5% | 19.3% | 4.6% | 3.9% | 13.8% |
|  | 2013 | 45.9% | 5.6% | 2.1% | 0.0% | 25.2% | 5.3% | 3.5% | 12.4% |
|  | 2014 | 45.8% | 7.2% | 4.1% | 0.0% | 22.1% | 7.4% | 2.0% | 11.4% |
|  | 2015 | 45.3% | 4.6% | 3.0% | 0.2% | 24.5% | 7.1% | 4.2% | 11.1% |
|  | 2016 | 42.7% | 4.8% | 4.3% | 0.0% | 25.6% | 6.0% | 2.9% | 13.8% |
|  | 2017 | 43.6% | 6.3% | 4.2% | 0.2% | 27.5% | 6.2% | 2.0% | 10.0% |
|  | 2018 | 38.4% | 4.3% | 3.8% | 0.0% | 29.2% | 7.2% | 4.5% | 12.5% |
|  | 2019 | 33.1% | 2.9% | 4.4% | 0.2% | 34.2% | 9.1% | 2.9% | 13.3% |
|  | 2020 | 36.0% | 4.0% | 2.4% | 0.0% | 33.0% | 10.7% | 1.6% | 12.3% |
| Regional | 2000 | 28.5% | 16.0% | 3.1% | 2.0% | 11.8% | 5.2% | 16.4% | 17.0% |
|  | 2001 | 27.9% | 16.3% | 2.8% | 1.7% | 12.5% | 5.7% | 16.5% | 16.5% |
|  | 2002 | 29.7% | 17.9% | 2.7% | 1.2% | 9.4% | 4.7% | 17.1% | 17.4% |
|  | 2003 | 31.6% | 14.0% | 2.4% | 1.6% | 11.7% | 3.4% | 13.0% | 22.2% |
|  | 2004 | 29.9% | 12.9% | 3.5% | 0.9% | 12.4% | 5.5% | 15.6% | 19.4% |
|  | 2005 | 29.9% | 13.2% | 1.8% | 1.1% | 14.7% | 6.6% | 13.8% | 18.9% |
|  | 2006 | 27.8% | 11.8% | 1.7% | 1.1% | 15.2% | 9.2% | 12.5% | 20.7% |
|  | 2007 | 27.2% | 9.8% | 2.3% | 1.2% | 16.7% | 8.7% | 12.7% | 21.5% |
|  | 2008 | 24.3% | 10.1% | 2.1% | 1.0% | 17.0% | 9.0% | 14.4% | 22.2% |
|  | 2009 | 25.4% | 9.4% | 2.1% | 0.9% | 19.7% | 11.0% | 11.1% | 20.3% |
|  | 2010 | 27.1% | 8.8% | 2.1% | 0.1% | 20.2% | 12.5% | 10.6% | 18.6% |
|  | 2011 | 27.4% | 7.7% | 1.5% | 0.5% | 21.1% | 11.5% | 11.9% | 18.3% |
|  | 2012 | 25.9% | 7.5% | 1.6% | 0.7% | 24.4% | 11.1% | 11.5% | 17.4% |
|  | 2013 | 24.7% | 7.3% | 1.8% | 0.2% | 26.3% | 10.9% | 11.6% | 17.3% |
|  | 2014 | 24.0% | 7.1% | 0.9% | 0.2% | 29.3% | 12.9% | 9.8% | 15.8% |
|  | 2015 | 25.1% | 6.7% | 1.8% | 0.1% | 29.4% | 12.5% | 9.8% | 14.6% |
|  | 2016 | 23.8% | 4.4% | 1.5% | 0.1% | 35.2% | 14.8% | 7.5% | 12.8% |
|  | 2017 | 23.6% | 5.2% | 1.2% | 0.1% | 35.0% | 14.6% | 6.5% | 13.8% |
|  | 2018 | 20.9% | 3.8% | 1.9% | 0.1% | 31.2% | 18.2% | 8.5% | 15.3% |
|  | 2019 | 19.9% | 4.3% | 1.4% | 0.0% | 34.3% | 17.6% | 7.6% | 15.0% |
|  | 2020 | 20.5% | 3.9% | 1.9% | 0.0% | 33.7% | 18.7% | 6.5% | 14.7% |
| Distant | 2000 | 50.0% | 3.6% | 2.2% | 0.3% | 34.3% | 2.8% | 1.2% | 5.4% |
|  | 2001 | 47.6% | 4.9% | 2.2% | 0.2% | 35.6% | 2.7% | 1.3% | 5.5% |
|  | 2002 | 47.2% | 4.6% | 1.7% | 0.2% | 37.5% | 3.0% | 1.4% | 4.4% |
|  | 2003 | 50.1% | 1.6% | 2.0% | 0.1% | 38.9% | 0.4% | 0.6% | 6.3% |
|  | 2004 | 50.3% | 1.4% | 1.7% | 0.0% | 38.3% | 1.1% | 0.9% | 6.2% |
|  | 2005 | 48.4% | 1.5% | 1.5% | 0.1% | 41.2% | 1.3% | 0.7% | 5.3% |
|  | 2006 | 48.0% | 1.0% | 1.9% | 0.1% | 41.7% | 1.4% | 1.0% | 4.8% |
|  | 2007 | 46.5% | 1.6% | 1.8% | 0.0% | 43.2% | 1.0% | 0.4% | 5.5% |
|  | 2008 | 46.1% | 1.4% | 1.3% | 0.0% | 43.8% | 1.4% | 0.6% | 5.4% |
|  | 2009 | 44.3% | 1.2% | 1.3% | 0.1% | 46.0% | 1.4% | 0.9% | 4.6% |
|  | 2010 | 45.7% | 0.7% | 1.5% | 0.0% | 45.1% | 1.2% | 0.7% | 5.2% |
|  | 2011 | 45.1% | 0.7% | 1.1% | 0.1% | 45.8% | 1.4% | 0.6% | 5.3% |
|  | 2012 | 43.4% | 1.1% | 1.5% | 0.0% | 48.0% | 1.2% | 0.5% | 4.3% |
|  | 2013 | 42.8% | 0.7% | 1.5% | 0.1% | 48.9% | 1.3% | 0.4% | 4.2% |
|  | 2014 | 44.3% | 0.7% | 1.5% | 0.0% | 48.7% | 1.2% | 0.3% | 3.2% |
|  | 2015 | 43.7% | 0.6% | 1.1% | 0.0% | 49.5% | 1.0% | 0.5% | 3.6% |
|  | 2016 | 42.7% | 0.3% | 1.4% | 0.0% | 51.1% | 1.0% | 0.3% | 3.1% |
|  | 2017 | 41.6% | 0.3% | 1.1% | 0.0% | 52.5% | 1.3% | 0.2% | 3.0% |
|  | 2018 | 42.4% | 0.6% | 1.2% | 0.0% | 50.6% | 1.2% | 0.3% | 3.7% |
|  | 2019 | 40.4% | 0.2% | 1.2% | 0.0% | 53.1% | 1.1% | 0.4% | 3.6% |
|  | 2020 | 41.1% | 0.5% | 0.9% | 0.0% | 52.9% | 1.0% | 0.3% | 3.2% |

**Supplementary Table S5.** Distribution of treatment in IPMN.

| Stage | Year of diagnosis | Treatment | | | | | | | |
| --- | --- | --- | --- | --- | --- | --- | --- | --- | --- |
|  |  | None | Surgery | Radiotherapy | Radiotherapy+Surgery | Chemotherapy | Chemotherapy+Surgery | Radiotherapy+Chemotherapy+Surgery | Chemotherapy+Radiotherapy |
| Overall | 2000 | 37.8% | 16.3% | 2.1% | 0.6% | 26.5% | 4.8% | 6.3% | 5.7% |
|  | 2001 | 37.8% | 15.1% | 1.8% | 2.4% | 21.8% | 5.1% | 9.0% | 7.0% |
|  | 2002 | 37.2% | 15.7% | 1.6% | 0.9% | 31.3% | 4.5% | 3.6% | 5.2% |
|  | 2003 | 43.9% | 7.8% | 3.5% | 0.0% | 28.4% | 1.9% | 6.3% | 8.1% |
|  | 2004 | 43.4% | 9.7% | 2.0% | 0.0% | 28.6% | 5.4% | 5.1% | 5.9% |
|  | 2005 | 40.0% | 9.8% | 2.2% | 1.4% | 27.6% | 6.6% | 3.8% | 8.5% |
|  | 2006 | 35.3% | 13.2% | 2.2% | 0.6% | 29.7% | 4.4% | 6.8% | 7.7% |
|  | 2007 | 40.5% | 10.6% | 2.6% | 1.3% | 29.5% | 3.9% | 6.5% | 5.2% |
|  | 2008 | 34.9% | 9.9% | 0.4% | 1.0% | 35.4% | 6.3% | 6.3% | 6.0% |
|  | 2009 | 39.2% | 10.6% | 2.2% | 0.8% | 30.2% | 5.3% | 6.5% | 5.2% |
|  | 2010 | 36.9% | 11.0% | 0.5% | 0.0% | 28.7% | 8.3% | 4.7% | 9.9% |
|  | 2011 | 31.5% | 12.9% | 1.3% | 0.9% | 30.7% | 8.9% | 5.7% | 8.2% |
|  | 2012 | 33.0% | 10.2% | 0.7% | 0.0% | 35.7% | 6.3% | 6.8% | 7.3% |
|  | 2013 | 31.9% | 9.5% | 0.8% | 0.0% | 39.2% | 10.0% | 2.8% | 5.8% |
|  | 2014 | 28.9% | 10.5% | 3.0% | 0.0% | 36.5% | 10.8% | 5.1% | 5.3% |
|  | 2015 | 28.7% | 13.4% | 1.9% | 0.5% | 35.7% | 9.2% | 6.2% | 4.4% |
|  | 2016 | 29.1% | 10.1% | 1.4% | 0.0% | 35.7% | 12.7% | 5.0% | 6.0% |
|  | 2017 | 30.7% | 7.5% | 1.2% | 0.0% | 38.6% | 14.6% | 3.5% | 3.8% |
|  | 2018 | 37.8% | 10.9% | 2.8% | 0.0% | 27.3% | 16.7% | 2.4% | 2.1% |
|  | 2019 | 23.5% | 15.3% | 1.1% | 0.0% | 39.4% | 13.5% | 1.7% | 5.5% |
|  | 2020 | 29.7% | 9.9% | 0.7% | 0.0% | 30.2% | 18.2% | 2.9% | 8.5% |
| Localized | 2000 | 30.4% | 53.1% | 0.0% | 0.0% | 8.1% | 0.0% | 4.0% | 4.4% |
|  | 2001 | 22.7% | 49.7% | 5.3% | 0.0% | 5.6% | 0.0% | 5.6% | 11.1% |
|  | 2002 | 33.4% | 48.9% | 0.0% | 0.0% | 0.0% | 0.0% | 8.7% | 9.0% |
|  | 2003 | 13.3% | 30.7% | 6.3% | 0.0% | 5.8% | 11.7% | 18.0% | 14.1% |
|  | 2004 | 27.5% | 31.9% | 4.4% | 0.0% | 5.1% | 9.4% | 7.6% | 14.1% |
|  | 2005 | 24.9% | 35.2% | 0.0% | 0.0% | 6.5% | 13.0% | 0.0% | 20.3% |
|  | 2006 | 16.4% | 54.0% | 0.0% | 0.0% | 6.0% | 0.0% | 17.3% | 6.4% |
|  | 2007 | 8.1% | 37.0% | 0.0% | 7.8% | 11.3% | 16.1% | 11.0% | 8.7% |
|  | 2008 | 22.2% | 43.1% | 0.0% | 2.9% | 3.5% | 13.3% | 8.7% | 6.4% |
|  | 2009 | 24.0% | 43.7% | 0.0% | 0.0% | 12.7% | 15.6% | 4.0% | 0.0% |
|  | 2010 | 26.5% | 42.3% | 0.0% | 0.0% | 3.9% | 27.3% | 0.0% | 0.0% |
|  | 2011 | 12.0% | 68.8% | 0.0% | 3.3% | 2.2% | 11.1% | 0.0% | 2.6% |
|  | 2012 | 49.4% | 29.5% | 3.5% | 0.0% | 2.9% | 2.9% | 4.0% | 7.7% |
|  | 2013 | 23.7% | 31.7% | 0.0% | 0.0% | 21.1% | 16.3% | 0.0% | 7.2% |
|  | 2014 | 22.9% | 34.6% | 0.0% | 0.0% | 13.1% | 16.5% | 13.0% | 0.0% |
|  | 2015 | 6.8% | 53.2% | 0.0% | 0.0% | 17.2% | 16.9% | 5.9% | 0.0% |
|  | 2016 | 14.3% | 26.6% | 0.0% | 0.0% | 15.0% | 28.4% | 4.4% | 11.3% |
|  | 2017 | 24.2% | 38.6% | 3.9% | 0.0% | 13.4% | 19.9% | 0.0% | 0.0% |
|  | 2018 | 18.6% | 36.5% | 2.3% | 0.0% | 12.2% | 30.3% | 0.0% | 0.0% |
|  | 2019 | 15.2% | 33.6% | 2.7% | 0.0% | 17.0% | 25.3% | 0.0% | 6.2% |
|  | 2020 | 18.5% | 38.1% | 3.3% | 0.0% | 16.5% | 23.7% | 0.0% | 0.0% |
| Regional | 2000 | 17.2% | 24.8% | 4.6% | 1.0% | 13.5% | 5.7% | 22.2% | 11.1% |
|  | 2001 | 21.1% | 20.5% | 1.3% | 3.8% | 7.6% | 3.9% | 29.1% | 12.8% |
|  | 2002 | 21.3% | 27.3% | 0.0% | 3.4% | 10.4% | 11.7% | 12.3% | 13.7% |
|  | 2003 | 34.4% | 15.0% | 3.7% | 0.0% | 10.8% | 3.3% | 19.3% | 13.5% |
|  | 2004 | 31.7% | 19.7% | 1.3% | 0.0% | 8.7% | 8.4% | 15.9% | 14.4% |
|  | 2005 | 26.3% | 20.5% | 1.1% | 4.0% | 11.0% | 10.8% | 13.2% | 12.9% |
|  | 2006 | 12.3% | 23.8% | 2.8% | 2.5% | 13.4% | 13.2% | 16.9% | 15.2% |
|  | 2007 | 27.0% | 23.0% | 0.0% | 2.0% | 10.7% | 7.3% | 19.1% | 10.9% |
|  | 2008 | 22.8% | 15.5% | 0.0% | 1.5% | 11.7% | 9.8% | 20.9% | 17.8% |
|  | 2009 | 20.6% | 22.0% | 0.0% | 1.7% | 12.6% | 11.0% | 19.2% | 12.7% |
|  | 2010 | 18.5% | 16.9% | 1.5% | 0.0% | 13.7% | 15.9% | 13.3% | 20.0% |
|  | 2011 | 25.9% | 10.1% | 1.0% | 1.5% | 10.8% | 18.1% | 16.8% | 15.8% |
|  | 2012 | 11.8% | 20.8% | 1.4% | 0.0% | 21.6% | 14.8% | 20.6% | 9.1% |
|  | 2013 | 26.2% | 17.9% | 1.4% | 0.0% | 19.1% | 21.2% | 8.2% | 6.1% |
|  | 2014 | 15.4% | 15.7% | 1.6% | 0.0% | 26.7% | 22.7% | 11.2% | 6.8% |
|  | 2015 | 17.7% | 11.4% | 0.0% | 1.5% | 24.1% | 20.5% | 16.0% | 8.7% |
|  | 2016 | 16.9% | 20.1% | 1.6% | 0.0% | 10.9% | 27.0% | 15.6% | 7.9% |
|  | 2017 | 14.2% | 6.5% | 2.9% | 0.0% | 18.1% | 40.1% | 9.1% | 9.1% |
|  | 2018 | 24.7% | 7.8% | 2.6% | 0.0% | 19.8% | 32.2% | 8.8% | 4.2% |
|  | 2019 | 5.8% | 26.0% | 2.2% | 0.0% | 19.6% | 32.2% | 6.7% | 7.4% |
|  | 2020 | 10.2% | 6.8% | 0.0% | 0.0% | 22.4% | 38.3% | 7.3% | 14.9% |
| Distant | 2000 | 45.7% | 8.6% | 1.4% | 0.5% | 35.4% | 5.2% | 0.0% | 3.3% |
|  | 2001 | 44.9% | 9.3% | 1.8% | 2.3% | 29.6% | 6.8% | 0.6% | 4.7% |
|  | 2002 | 40.7% | 9.5% | 2.3% | 0.4% | 41.5% | 3.2% | 1.0% | 1.4% |
|  | 2003 | 48.9% | 2.2% | 2.8% | 0.0% | 39.7% | 0.6% | 0.0% | 5.8% |
|  | 2004 | 49.4% | 2.7% | 2.1% | 0.0% | 40.0% | 3.8% | 0.5% | 1.5% |
|  | 2005 | 45.7% | 3.5% | 3.0% | 0.6% | 36.4% | 4.5% | 0.5% | 6.0% |
|  | 2006 | 46.3% | 2.5% | 2.3% | 0.0% | 40.0% | 2.0% | 1.5% | 5.3% |
|  | 2007 | 49.4% | 1.3% | 4.2% | 0.0% | 41.8% | 0.7% | 0.7% | 1.9% |
|  | 2008 | 40.9% | 2.1% | 0.6% | 0.5% | 50.4% | 4.1% | 0.0% | 1.5% |
|  | 2009 | 47.4% | 1.3% | 3.5% | 0.5% | 41.2% | 1.5% | 1.6% | 2.9% |
|  | 2010 | 48.4% | 3.0% | 0.0% | 0.0% | 40.6% | 1.3% | 0.7% | 6.0% |
|  | 2011 | 38.3% | 0.9% | 1.9% | 0.0% | 49.7% | 3.6% | 1.1% | 4.4% |
|  | 2012 | 37.7% | 1.8% | 0.0% | 0.0% | 49.0% | 3.4% | 1.4% | 6.7% |
|  | 2013 | 35.4% | 0.9% | 0.8% | 0.0% | 52.0% | 4.2% | 1.1% | 5.6% |
|  | 2014 | 36.4% | 2.3% | 4.5% | 0.0% | 47.9% | 3.1% | 0.0% | 5.7% |
|  | 2015 | 41.1% | 3.3% | 3.4% | 0.0% | 46.7% | 1.3% | 1.0% | 3.2% |
|  | 2016 | 37.8% | 1.5% | 1.7% | 0.0% | 53.0% | 2.2% | 0.0% | 3.9% |
|  | 2017 | 37.6% | 2.0% | 0.0% | 0.0% | 53.3% | 3.0% | 1.9% | 2.3% |
|  | 2018 | 52.1% | 2.1% | 3.2% | 0.0% | 38.0% | 2.8% | 0.0% | 1.8% |
|  | 2019 | 35.2% | 1.3% | 0.0% | 0.0% | 59.1% | 0.0% | 0.0% | 4.4% |
|  | 2020 | 47.1% | 0.0% | 0.0% | 0.0% | 41.8% | 2.2% | 1.1% | 7.7% |

**Supplementary Table S6.** Distribution of treatment in pNET.

| Stage | Year of diagnosis | Treatment | | | | | | | |
| --- | --- | --- | --- | --- | --- | --- | --- | --- | --- |
|  |  | None | Surgery | Radiotherapy | Radiotherapy+Surgery | Chemotherapy | Chemotherapy+Surgery | Radiotherapy+Chemotherapy+Surgery | Chemotherapy+Radiotherapy |
| Overall | 2000 | 39.8% | 31.0% | 0.7% | 2.2% | 16.8% | 5.7% | 0.7% | 3.0% |
|  | 2001 | 32.8% | 35.7% | 1.9% | 0.7% | 21.5% | 3.6% | 2.5% | 1.3% |
|  | 2002 | 35.6% | 30.0% | 2.2% | 1.1% | 19.9% | 7.4% | 0.6% | 3.2% |
|  | 2003 | 36.2% | 29.0% | 0.6% | 1.0% | 22.9% | 6.0% | 0.5% | 3.9% |
|  | 2004 | 32.5% | 30.4% | 2.5% | 0.0% | 23.8% | 4.0% | 3.0% | 3.8% |
|  | 2005 | 33.6% | 30.1% | 3.9% | 0.3% | 21.2% | 6.2% | 0.9% | 3.8% |
|  | 2006 | 30.1% | 35.2% | 2.5% | 0.8% | 22.4% | 6.5% | 1.7% | 0.8% |
|  | 2007 | 33.4% | 34.0% | 3.5% | 0.0% | 21.7% | 2.5% | 1.1% | 3.7% |
|  | 2008 | 25.8% | 42.4% | 1.0% | 0.4% | 19.0% | 7.4% | 0.8% | 3.2% |
|  | 2009 | 32.2% | 39.6% | 3.0% | 0.3% | 18.3% | 2.5% | 1.1% | 2.9% |
|  | 2010 | 25.6% | 39.9% | 1.6% | 0.5% | 21.7% | 5.9% | 1.1% | 3.7% |
|  | 2011 | 26.6% | 42.6% | 1.1% | 0.6% | 21.5% | 4.3% | 0.7% | 2.5% |
|  | 2012 | 19.7% | 52.7% | 0.2% | 1.0% | 19.1% | 4.6% | 0.7% | 1.9% |
|  | 2013 | 26.0% | 50.4% | 1.3% | 0.5% | 16.5% | 3.1% | 0.8% | 1.5% |
|  | 2014 | 23.2% | 51.8% | 1.5% | 0.8% | 17.6% | 2.2% | 0.4% | 2.6% |
|  | 2015 | 23.2% | 54.1% | 0.7% | 0.4% | 16.0% | 3.6% | 0.8% | 1.4% |
|  | 2016 | 24.1% | 54.2% | 1.3% | 0.4% | 15.0% | 2.6% | 0.6% | 1.8% |
|  | 2017 | 26.4% | 54.9% | 0.6% | 0.0% | 14.3% | 2.6% | 0.0% | 1.2% |
|  | 2018 | 32.2% | 49.8% | 1.3% | 0.4% | 11.7% | 2.3% | 0.4% | 1.8% |
|  | 2019 | 33.4% | 48.0% | 1.1% | 0.3% | 12.5% | 3.1% | 0.2% | 1.4% |
|  | 2020 | 36.5% | 46.4% | 1.2% | 0.3% | 12.4% | 1.4% | 0.4% | 1.3% |
| Localized | 2000 | 10.7% | 84.0% | 0.0% | 0.0% | 0.0% | 0.0% | 0.0% | 5.3% |
|  | 2001 | 5.5% | 94.5% | 0.0% | 0.0% | 0.0% | 0.0% | 0.0% | 0.0% |
|  | 2002 | 8.8% | 76.6% | 9.6% | 0.0% | 0.0% | 5.1% | 0.0% | 0.0% |
|  | 2003 | 17.5% | 82.5% | 0.0% | 0.0% | 0.0% | 0.0% | 0.0% | 0.0% |
|  | 2004 | 34.5% | 65.5% | 0.0% | 0.0% | 0.0% | 0.0% | 0.0% | 0.0% |
|  | 2005 | 30.5% | 64.2% | 0.0% | 0.0% | 0.0% | 2.6% | 0.0% | 2.6% |
|  | 2006 | 15.4% | 79.8% | 2.2% | 0.0% | 0.0% | 0.0% | 2.6% | 0.0% |
|  | 2007 | 7.7% | 90.1% | 2.2% | 0.0% | 0.0% | 0.0% | 0.0% | 0.0% |
|  | 2008 | 18.5% | 78.2% | 3.3% | 0.0% | 0.0% | 0.0% | 0.0% | 0.0% |
|  | 2009 | 12.3% | 86.0% | 0.0% | 0.0% | 1.7% | 0.0% | 0.0% | 0.0% |
|  | 2010 | 15.9% | 82.4% | 0.0% | 0.0% | 0.9% | 0.8% | 0.0% | 0.0% |
|  | 2011 | 21.2% | 76.2% | 0.0% | 0.0% | 1.9% | 0.8% | 0.0% | 0.0% |
|  | 2012 | 10.4% | 87.3% | 0.6% | 0.4% | 0.7% | 0.6% | 0.0% | 0.0% |
|  | 2013 | 17.2% | 80.3% | 0.9% | 0.0% | 0.4% | 0.7% | 0.5% | 0.0% |
|  | 2014 | 20.0% | 78.1% | 0.0% | 0.8% | 0.3% | 0.8% | 0.0% | 0.0% |
|  | 2015 | 16.5% | 81.5% | 0.0% | 0.5% | 1.3% | 0.3% | 0.0% | 0.0% |
|  | 2016 | 16.9% | 80.8% | 0.7% | 0.0% | 0.6% | 1.0% | 0.0% | 0.0% |
|  | 2017 | 23.8% | 74.7% | 0.3% | 0.0% | 1.0% | 0.2% | 0.0% | 0.0% |
|  | 2018 | 32.2% | 66.9% | 0.0% | 0.3% | 0.0% | 0.6% | 0.0% | 0.0% |
|  | 2019 | 33.4% | 64.7% | 0.3% | 0.3% | 0.3% | 0.8% | 0.0% | 0.2% |
|  | 2020 | 32.3% | 65.8% | 0.7% | 0.0% | 1.0% | 0.2% | 0.0% | 0.0% |
| Regional | 2000 | 24.6% | 55.9% | 0.0% | 7.9% | 0.0% | 11.7% | 0.0% | 0.0% |
|  | 2001 | 3.5% | 73.7% | 0.0% | 4.0% | 7.3% | 0.0% | 11.5% | 0.0% |
|  | 2002 | 6.7% | 74.2% | 0.0% | 0.0% | 0.0% | 12.7% | 3.5% | 3.0% |
|  | 2003 | 6.7% | 70.7% | 3.6% | 2.9% | 0.0% | 6.1% | 3.0% | 7.0% |
|  | 2004 | 13.4% | 48.7% | 0.0% | 0.0% | 18.7% | 5.7% | 10.7% | 2.8% |
|  | 2005 | 12.2% | 52.2% | 4.7% | 0.0% | 13.8% | 9.2% | 2.8% | 5.1% |
|  | 2006 | 12.6% | 66.7% | 0.0% | 3.9% | 7.7% | 7.5% | 0.0% | 1.7% |
|  | 2007 | 18.0% | 63.7% | 4.2% | 0.0% | 3.9% | 6.2% | 3.9% | 0.0% |
|  | 2008 | 9.6% | 67.2% | 0.0% | 0.0% | 4.6% | 10.7% | 3.3% | 4.5% |
|  | 2009 | 20.1% | 60.0% | 2.4% | 1.5% | 4.1% | 4.6% | 1.6% | 5.7% |
|  | 2010 | 10.1% | 65.3% | 2.6% | 1.4% | 6.9% | 7.9% | 4.2% | 1.8% |
|  | 2011 | 13.3% | 76.5% | 1.0% | 1.8% | 3.5% | 2.9% | 1.0% | 0.0% |
|  | 2012 | 12.9% | 71.9% | 0.0% | 2.9% | 4.7% | 5.0% | 2.1% | 0.7% |
|  | 2013 | 12.1% | 72.7% | 0.0% | 0.8% | 6.0% | 6.3% | 1.3% | 0.7% |
|  | 2014 | 9.7% | 74.4% | 0.7% | 0.0% | 7.9% | 3.7% | 0.9% | 2.6% |
|  | 2015 | 10.6% | 70.0% | 0.8% | 0.0% | 6.5% | 8.8% | 1.7% | 1.7% |
|  | 2016 | 7.7% | 74.2% | 1.5% | 2.0% | 8.2% | 4.3% | 1.1% | 0.9% |
|  | 2017 | 12.2% | 79.6% | 0.0% | 0.0% | 3.6% | 4.6% | 0.0% | 0.0% |
|  | 2018 | 12.4% | 77.4% | 0.0% | 0.0% | 5.4% | 2.1% | 0.7% | 2.0% |
|  | 2019 | 13.3% | 71.7% | 0.7% | 0.0% | 9.4% | 3.0% | 0.0% | 2.0% |
|  | 2020 | 20.3% | 63.2% | 0.0% | 0.8% | 9.7% | 2.3% | 1.8% | 1.9% |
| Distant | 2000 | 48.0% | 10.4% | 1.2% | 1.2% | 27.9% | 6.1% | 1.2% | 3.8% |
|  | 2001 | 42.1% | 16.1% | 3.1% | 0.0% | 30.1% | 5.7% | 0.9% | 2.0% |
|  | 2002 | 44.0% | 12.1% | 1.6% | 0.8% | 29.9% | 7.2% | 0.0% | 4.3% |
|  | 2003 | 44.2% | 9.2% | 0.0% | 0.8% | 34.2% | 8.1% | 0.0% | 3.5% |
|  | 2004 | 37.9% | 13.7% | 4.1% | 0.0% | 32.4% | 4.9% | 1.6% | 5.4% |
|  | 2005 | 40.6% | 12.5% | 5.0% | 0.5% | 30.3% | 6.6% | 0.6% | 3.9% |
|  | 2006 | 39.9% | 11.1% | 3.6% | 0.0% | 34.5% | 7.9% | 2.1% | 0.8% |
|  | 2007 | 43.3% | 13.4% | 3.2% | 0.0% | 32.4% | 2.3% | 0.7% | 4.8% |
|  | 2008 | 38.1% | 16.2% | 0.0% | 0.8% | 31.1% | 9.6% | 0.0% | 4.1% |
|  | 2009 | 44.3% | 13.2% | 4.7% | 0.0% | 30.4% | 3.1% | 1.5% | 2.8% |
|  | 2010 | 34.7% | 11.1% | 2.1% | 0.5% | 37.6% | 7.5% | 0.6% | 5.9% |
|  | 2011 | 33.6% | 9.5% | 1.9% | 0.4% | 41.2% | 7.2% | 1.0% | 5.2% |
|  | 2012 | 28.1% | 13.9% | 0.0% | 0.6% | 44.3% | 8.1% | 0.6% | 4.5% |
|  | 2013 | 39.9% | 10.0% | 2.4% | 0.9% | 38.4% | 4.0% | 1.0% | 3.4% |
|  | 2014 | 29.7% | 12.5% | 3.6% | 1.2% | 43.2% | 3.2% | 0.7% | 5.8% |
|  | 2015 | 38.8% | 8.2% | 1.6% | 0.5% | 41.4% | 5.0% | 1.3% | 3.2% |
|  | 2016 | 39.8% | 10.0% | 1.9% | 0.0% | 38.5% | 3.8% | 1.0% | 4.9% |
|  | 2017 | 35.5% | 14.8% | 1.5% | 0.0% | 39.8% | 4.8% | 0.0% | 3.7% |
|  | 2018 | 39.8% | 12.8% | 4.0% | 0.7% | 32.2% | 5.0% | 0.9% | 4.5% |
|  | 2019 | 41.0% | 11.6% | 2.8% | 0.4% | 33.5% | 7.1% | 0.5% | 3.2% |
|  | 2020 | 46.9% | 12.3% | 2.5% | 0.5% | 31.3% | 3.0% | 0.4% | 3.1% |
