# Supplementary figures and images for: Trends in pancreatic cancer incidence, prevalence, and survival outcomes by histological subtypes: a retrospective cohort study
Source: Gastroenterol Rep (Oxf). 2025 Apr 9;13:goaf030. doi: 10.1093/gastro/goaf030 (PMC11981714; doi:10.1093/gastro/goaf030)

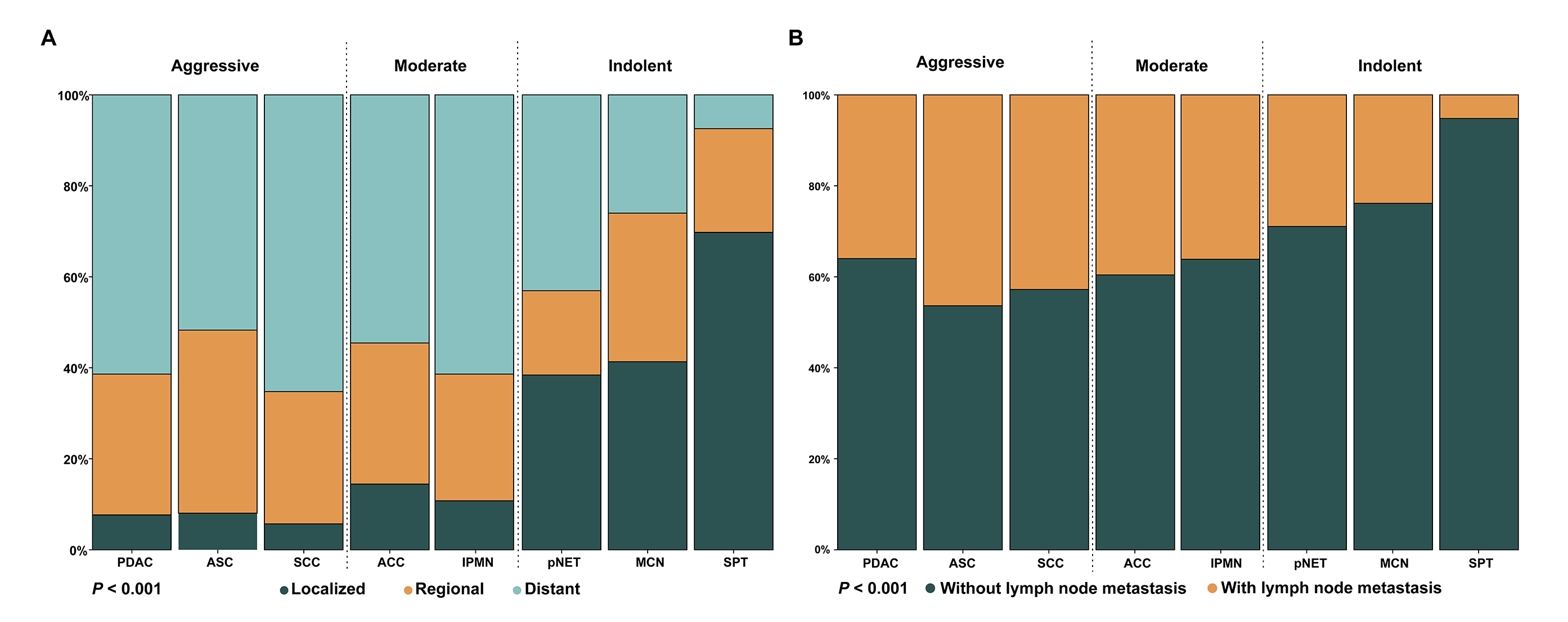

Supplement: goaf030_Supplementary_Data [file goaf030_supplementary_data.zip › Supplementary_Figure_S6.tif]

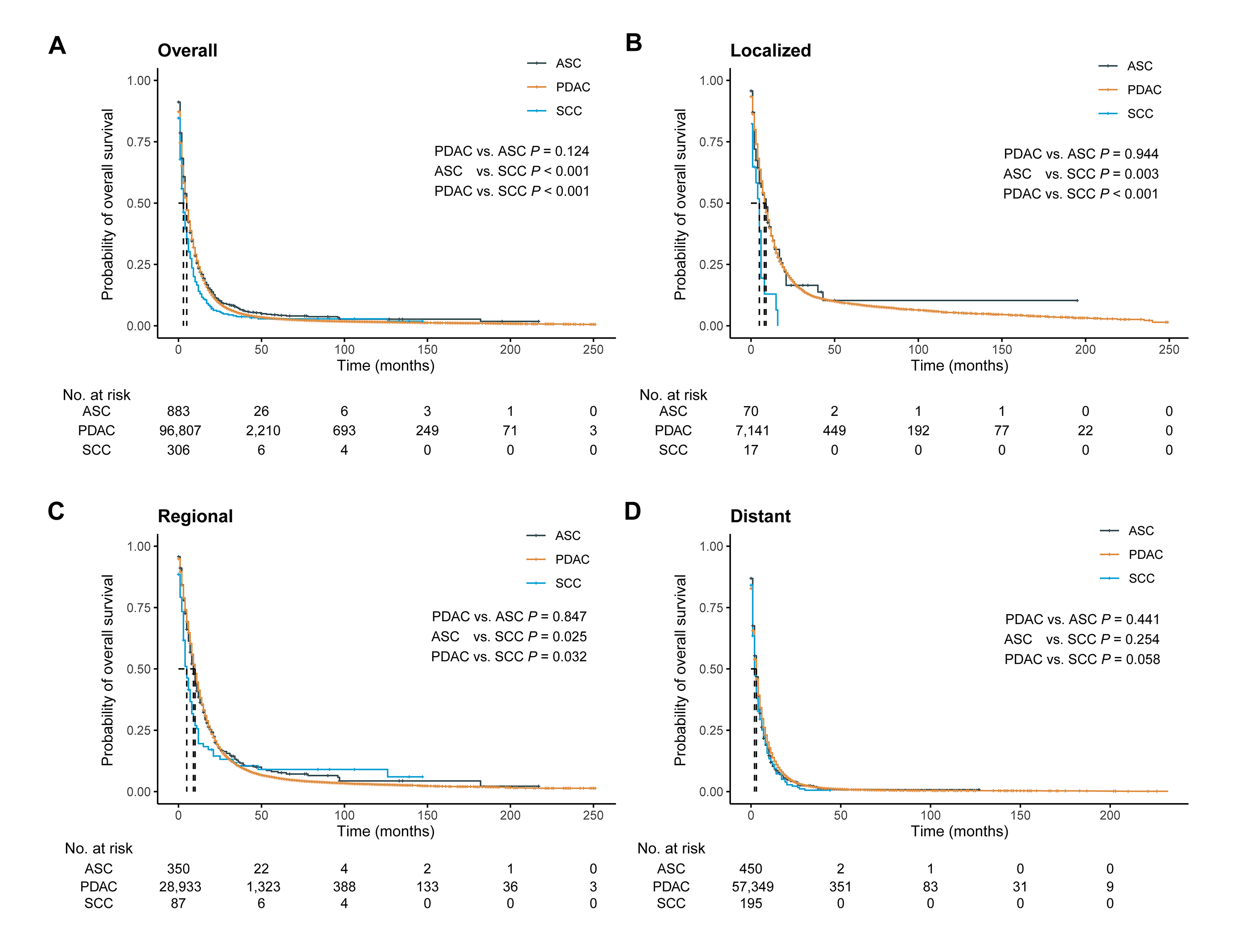

Supplement: goaf030_Supplementary_Data [file goaf030_supplementary_data.zip › Supplementary_Figure_S5.tif]

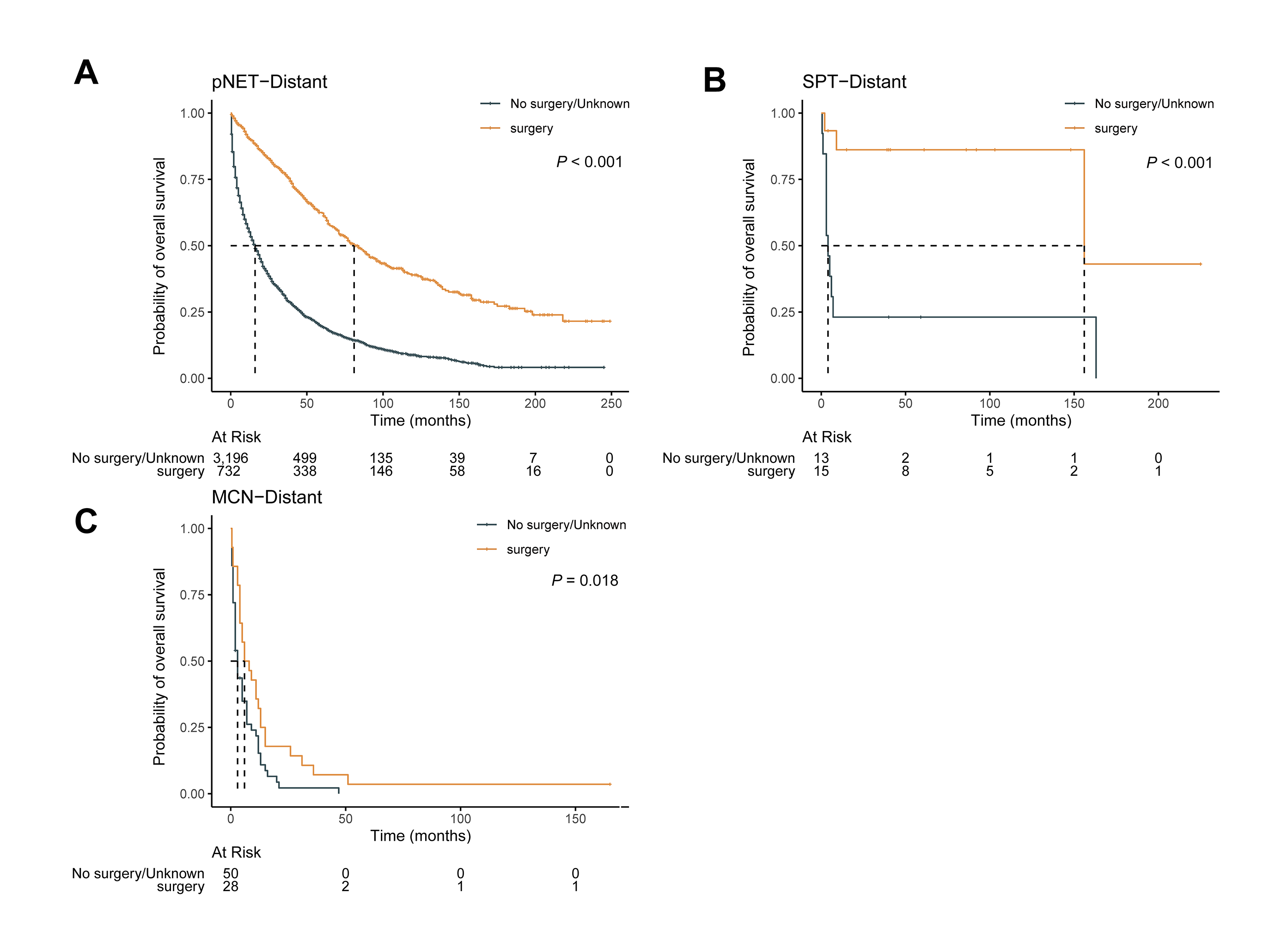

Supplement: goaf030_Supplementary_Data [file goaf030_supplementary_data.zip › Supplementary_Figure_S7.tif]

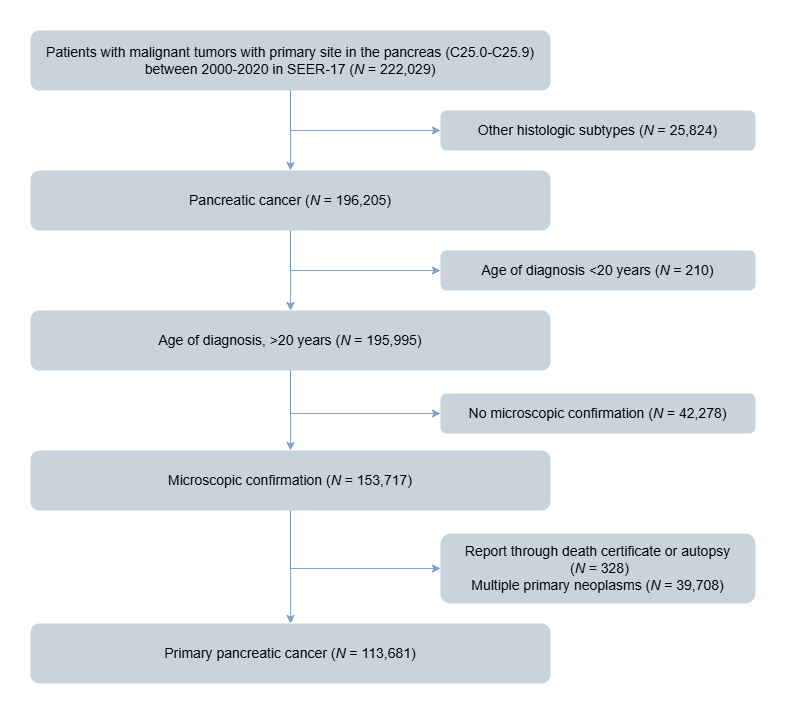

Supplement: goaf030_Supplementary_Data [file goaf030_supplementary_data.zip › Supplementary_Figure_S1.png]

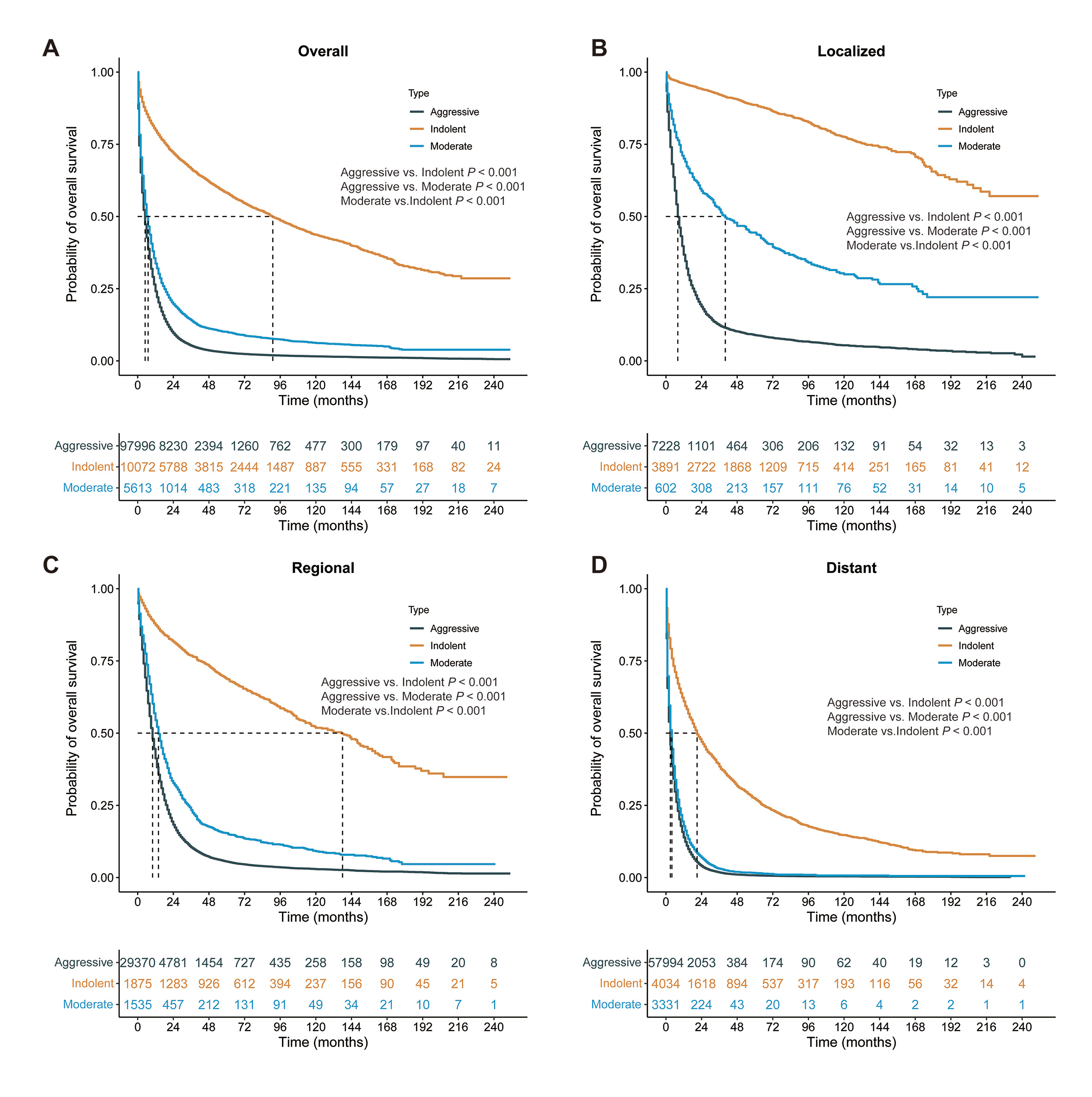

Supplement: goaf030_Supplementary_Data [file goaf030_supplementary_data.zip › Supplementary_Figure_S4.tif]

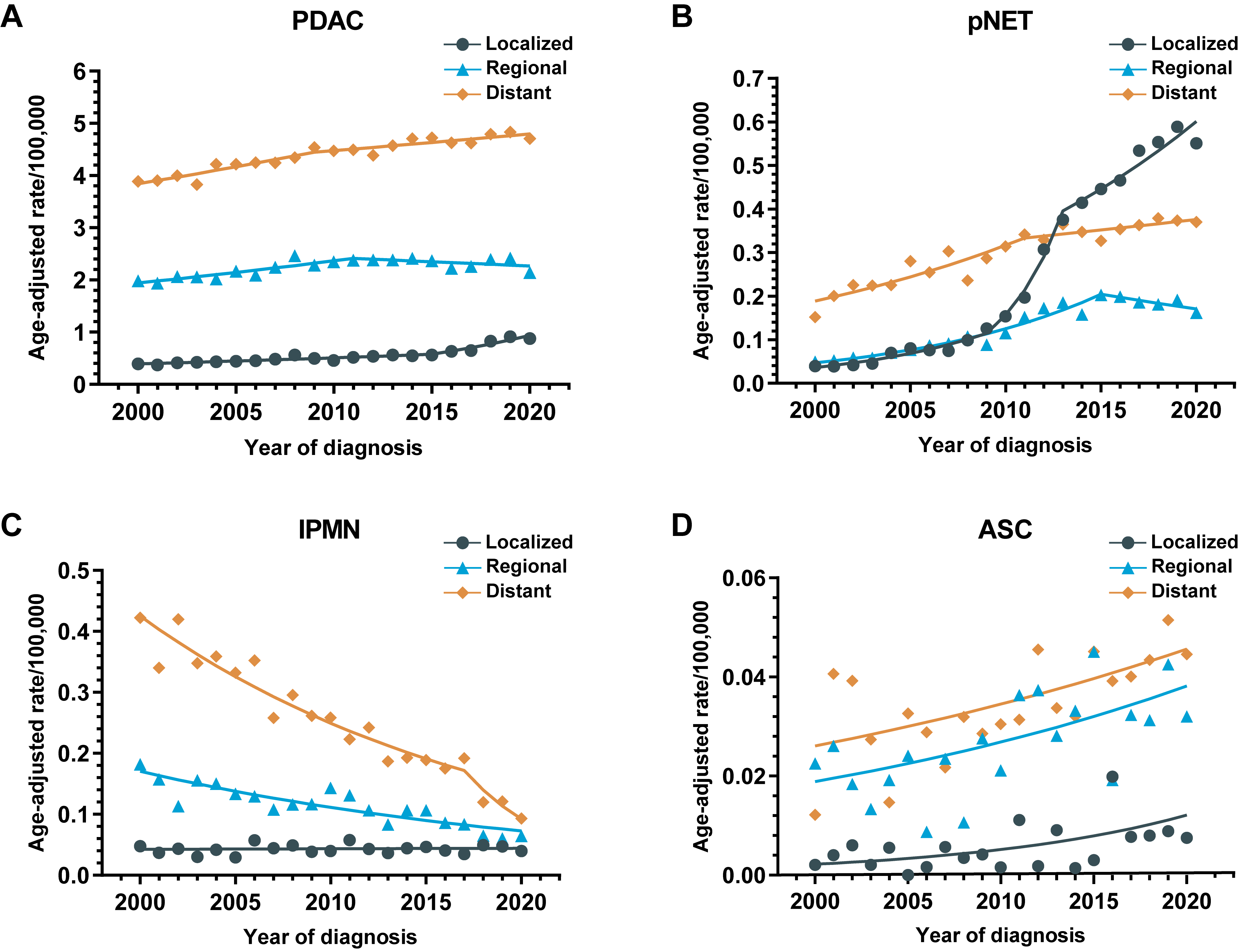

Supplement: goaf030_Supplementary_Data [file goaf030_supplementary_data.zip › Supplementary_Figure_S3.tif]
